# Expert workshop summary: Advancing toward a standardized murine model to evaluate treatments for antimicrobial resistance lung infections

**DOI:** 10.3389/fmicb.2022.988725

**Published:** 2022-09-08

**Authors:** Rakel Arrazuria, Bernhard Kerscher, Karen E. Huber, Jennifer L. Hoover, Carina Vingsbo Lundberg, Jon Ulf Hansen, Sylvie Sordello, Stephane Renard, Vincent Aranzana-Climent, Diarmaid Hughes, Philip Gribbon, Lena E. Friberg, Isabelle Bekeredjian-Ding

**Affiliations:** ^1^Division of Microbiology, Paul-Ehrlich-Institut, Langen, Germany; ^2^Infectious Diseases Research Unit, GlaxoSmithKline Pharmaceuticals, Collegeville, PA, United States; ^3^Department of Bacteria, Parasites & Fungi, Statens Serum Institut, Copenhagen, Denmark; ^4^Infectious Diseases, Evotec, Toulouse, France; ^5^Department of Pharmacy, Uppsala University, Uppsala, Sweden; ^6^Department of Medical Biochemistry and Microbiology, Uppsala University, Uppsala, Sweden; ^7^Fraunhofer Institute for Translational Medicine and Pharmacology ITMP, Discovery Research ScreeningPort, Hamburg, Germany; ^8^Institute of Medical Microbiology, Immunology and Parasitology, University Hospital Bonn, Bonn, Germany

**Keywords:** murine pneumonia model, antimicrobial, lung infection, Gram-negative, PK/PD, antimicrobial efficacy studies

## Abstract

The rise in antimicrobial resistance (AMR), and increase in treatment-refractory AMR infections, generates an urgent need to accelerate the discovery and development of novel anti-infectives. Preclinical animal models play a crucial role in assessing the efficacy of novel drugs, informing human dosing regimens and progressing drug candidates into the clinic. The Innovative Medicines Initiative-funded “Collaboration for prevention and treatment of MDR bacterial infections” (COMBINE) consortium is establishing a validated and globally harmonized preclinical model to increase reproducibility and more reliably translate results from animals to humans. Toward this goal, in April 2021, COMBINE organized the expert workshop “Advancing toward a standardized murine model to evaluate treatments for AMR lung infections”. This workshop explored the conduct and interpretation of mouse infection models, with presentations on PK/PD and efficacy studies of small molecule antibiotics, combination treatments (β-lactam/β-lactamase inhibitor), bacteriophage therapy, monoclonal antibodies and iron sequestering molecules, with a focus on the major Gram-negative AMR respiratory pathogens *Pseudomonas aeruginosa, Klebsiella pneumoniae* and *Acinetobacter baumannii.* Here we summarize the factors of variability that we identified in murine lung infection models used for antimicrobial efficacy testing, as well as the workshop presentations, panel discussions and the survey results for the harmonization of key experimental parameters. The resulting recommendations for standard design parameters are presented in this document and will provide the basis for the development of a harmonized and bench-marked efficacy studies in preclinical murine pneumonia model.

## Introduction

The loss of antibiotics as an effective tool to treat infections due to increasing antimicrobial resistance (AMR) is a serious threat to global health (Shallcross et al., 2015). For many patients suffering from these resistant infections, the danger of a post-antibiotic era has already become a devastating reality (Pourmand et al., 2017; Murray et al., 2022). Hence, there is a need for the accelerated development of new agents to treat and prevent infections caused by AMR pathogens (WHO, 2021). Despite increasing interest in the development of new or alternative therapies, there is a high attrition rate, and new therapies that often fail to reach the market (Hughes and Karlén, 2014; Bekeredjian-Ding, 2020).

The “Collaboration for prevention and treatment of MDR bacterial infections” (COMBINE) project is part of the European Innovative Medicines Initiative (IMI) Antimicrobial Resistance (AMR) Accelerator. The goal of the Accelerator is to progress the development of new medicines to treat or even prevent resistant bacterial infections. Preclinical efficacy models play a crucial role in the proof-of-concept efficacy investigations and provide the basis for selection of dosing regimens in clinical applications (Tängdén et al., 2020; Friberg, 2021). Differences in the commonly used preclinical models are extensive (Andes and Craig, 2002; Bulitta et al., 2019; Waack et al., 2020), limiting the results’ comparability and reproducibility and possibly impeding successful translation to the clinic. The pathogenesis of mouse pneumonia may have characteristics with those of human pneumonia despite anatomical and physiological differences (Mizgerd and Skerrett, 2008; Metersky and Waterer, 2020). However, pathogen-specific characteristics of virulence, infection route, infectious dose, and additional factors such animal genetic background all play a significant role in the pathology that is observed in mice (Mizgerd and Skerrett, 2008; Bielen et al., 2017; Dietert et al., 2017). To facilitate bench-to-bedside translation, and to accelerate and support the development of new antibiotics, it is necessary to establish reliable and globally harmonized preclinical models. Therefore, one of the scientific aims of COMBINE is the development of a standardized, validated murine model for the preclinical efficacy testing of novel anti-infective candidates.

We organized an expert workshop on April 27th and 28th, 2021 to discuss critical parameters of lung infection models conducted with the major Gram-negative AMR pathogens- *Pseudomonas aeruginosa, Klebsiella pneumoniae* and *Acinetobacter baumannii*. On the first day of the workshop, we shared our findings from a literature review of such models (accompanying review article: Variability of murine bacterial pneumonia models used to evaluate antimicrobial agents), presented a list of key variables and proposed standards to harmonize. Following discussion of the proposals in an expert panel forum and a survey of the workshop participants, we develop recommendations for efficacy studies (Table 1). The Supplementary material contains an overview of the second day of the workshop, we explored further applications of murine lung model such us bacteriophage therapy, monoclonal antibodies and iron sequestering molecules (Supplementary Figure 1 and Text).

**TABLE 1:** Summary of standard variables proposed by COMBINE experts, panel discussion and survey outcome.

Variable	Proposed parameter	Outcome of the expert discussion	Survey outcome	Comments and suggestions
**Animals**				
Mouse strain	CD-1 (outbred mice)	CD-1 (outbred mice)	CD-1 (outbred mice)	
Sex	Female	Female	Animals of both sexes	Confirmation of the results in the other gender may be necessary
Age or weight	6 weeks old	6 weeks old	6 weeks old	Further agreement in using 6-8 weeks mice at the start of any intervention
Number of animals per treatment	5-6 mice per treatment group	5-6 mice per treatment group	5-6 mice per treatment group	Adjust to the power analysis if necessary
Other		Create a best practice guideline		Animals from the same vendor. A minimum of acclimatization period. Randomization and blinding
**Inoculum**				
Source of strains	Include one *in vivo* validated strain from an accessible strain bank	Include one *in vivo* validated strain from an accessible strain bank	Include one strain from an accessible strain bank	
Culture media	Not standardized	Not standardized	Fresh bacterial culture media	Ensure inoculum viability and growth consistency
Growth stage	Log. Phase	Log. phase	Log. phase	
Inoculum preparation	Not standardized, but should ensure viability	Not standardized	Perform a washing step and use cold PBS as vehicle.	Minimize time between inoculum preparation and infection procedure
**Infection Procedure**				
Immunosuppression	Yes	Yes	Yes	
Cyclophosphamide protocol	150 mg/kg at -4 d and 100 mg/kg at -1 d	150 mg/kg at -4 d and 100 mg/kg at -1 d	150 mg/kg at -4 d and 100 mg/kg at -1 d	
Anesthesia	Not standardized	Not standardized	NA	Deep enough to allow the inoculum to settle in the lungs.
Infection route	IN	IN	IN	IT route may be considered for less pathogenic strains in mice
Infection volume	50 μL	20-50 μL	50 μL	
Inoculum	Not standardized	Not standardized	Not standardized	Not standardized considering baseline levels are standardized
**Treatment and Endpoints**				
Time to start of treatment	2 h p.i.	2 h p.i.	2 h p.i.	
Baseline CFU^1^	6.5-7.0 log_10_ CFU	6-7 log_10_ CFU	6.5-7.0 log_10_ CFU	
Min. CFU growth in untreated mice	1 log_10_ CFU/lung	1 log_10_ CFU/lung	1 log_10_ CFU/lung	
Length of study	26 h p.i.	26 h p.i.	26 h p.i.	Take several time points including 26 h p.i. if needed
Primary endpoint	CFU/lung	CFU/lung	CFU/lung	
Sample processing methods	Not standardized	Not standardized	Not standardized	Handle all samples from a study the same

NA: Not available, d: day, p.i.: post infection, IN: Intranasal,

^1^: Baseline CFU at the start of treatment, Min.: Minimum.

## Panel discussion on standardization of the acute murine pneumonia model for PK-PD and efficacy studies of small molecule antibiotics

**FIGURE 1:**
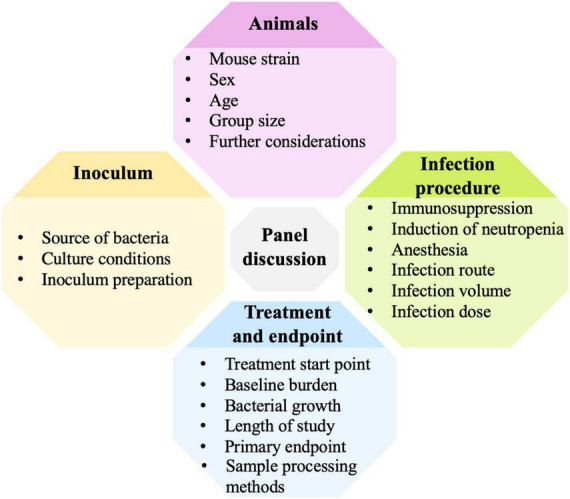
Murine pneumonia model variables addressed in the panel discussion.

A panel discussion with experts from academia, government and the pharmaceutical industry was held on the first day of the workshop (Supplementary Figure 1). This was organized into four different sections: (1) the animal, (2) the inoculum, (3) the infection procedure, and (4) the treatment and endpoints (Figure 1). The COMBINE experts presented a list of previously identified factors of variability and provided a proposed standard for each variable. This was followed by an open panel discussion, with the aim of reaching consensus on the parameters for inclusion in our standardized preclinical murine pneumonia model for efficacy studies of small-molecule antibiotics. The following summarize the panel’s and participants discussion.

### Animal variables

*Mouse strain:* CD1 outbred mice are commonly used in PK/PD testing (Bulitta et al., 2019); therefore, this was proposed as the standard. The three bacterial species of primary interest (*P. aeruginosa, K. pneumoniae* and *A. baumannii*) have all been shown to have good infectivity in this mouse strain. In addition, CD1 mice are outbred and thus less expensive than many inbred strains. CD1 mice are also the strain of choice for the thigh infection model and use of the same mouse strain allows for a better comparison between these two commonly used murine models. The panel recognized that the use of inbred mice may be advantageous under some circumstances, including for bacterial strains that are less virulent and show greater consistency in establishing infection.

*Sex:* Female mice are predominantly used in murine pneumonia models, most likely because their behavior is generally more amenable to group housing (Jennings et al., 1998). Some studies describe sex differences in the susceptibility to infection, for example with *A. baumannii* (Pires et al., 2020), and sex differences have also been described in PK (Soldin and Mattison, 2009; Madla et al., 2021). Our recommendation is to use female mice, consistent with the overall preference, accepting that this may neglect putative sex differences. A rationale for conducting studies in a single sex of mice (instead of both males and females) may be needed since regulatory agencies may encourage studies in both sexes. If confirmation of results in the other sex is deemed necessary, the extent of duplicative work should be balanced against the ethical considerations of using additional animals for preclinical experimentation. In these cases, only bridging studies should be considered.

*Age:* We propose the use of young or juvenile outbred animals of at least 6 weeks of age at the time of any intervention start. This is the most common age used based on a literature analysis. When working with inbred mice, eight weeks of age or older is preferred due to their slower growth and to ensure animals display a mature immune system. The experts noted that a random allocation based on the body weight should be applied to have consistent groups if smaller- or larger-than-average animals of the same age are included in the same group.

*Group size:* The number of animals per group used in preclinical studies are based on the power analysis for a given effect size. Acute lung infection models, typically use five to six animals per group, thus these numbers were proposed as the standard for 24-h efficacy studies with small molecule antibiotics. However, the number of animals may need to be adjusted based on the results of a statistical power analysis. Additionally, for survival studies and/or chronic infection models with high expected variability, no less than 10 mice per group are usually required.

*Further considerations:* Additional animal-related variables may impact the study outcome (vendor, acclimatization, randomization, enrichment in the cage, microbiota), but their standardization was not considered feasible due to differences in the established or approved practices at individual institutions or the regional location of the facilities. To capture these important considerations, the creation of a recommended best practices guideline along with the standard murine pneumonia protocol was suggested.

### Inoculum variables

#### Bacterial strains

It is well known that dose-response relationships can vary by bacterial strain, and this should be considered carefully when designing and conducting antimicrobial efficacy studies. Clinical strains differ in terms of the source, maintenance, number of passages, etc. For benchmarking or comparison of the results, which can be a powerful means of demonstrating the validity of the data, it was recommended to include at least one *in vivo* validated isolate (previously tested in mice and with at least 1 log_10_ growth in lung between 2h to 26 h p.i.), per bacterial species that is easily and globally accessible. While some labs routinely passage isolates in animals to boost virulence, this is not a common practice. Due to the possibility of genetic drift, we do not advocate clinical strains being passed on to animals.

#### Culture media

Different solid and liquid culture media may be used to grow the bacterial inoculum prior to infection of the mice. Although the majority of the institutions use broth culture, some groups use agar culture, subculturing the stocks overnight on agar media and suspending the bacteria in saline for inoculation. Each institution has their own standard procedures for bacterial culture; the consensus opinion was that the standardization of the type of media used was not necessary, as long as the bacteria are in log-phase growth, bacteria load baseline requirements are met and there is a consistent growth pattern.

#### Inoculum preparation

Similar to the culture media, the methods used for inoculum preparation are well established within each research group and vary depending on the institution. Standardizing the preparation of the inoculum was not considered necessary as long as reproducible bacterial viability is ensured. All the experts agreed that having consistency in the baseline count from the animal (discussed in treatment and endpoint variables section) is key for model reproducibility. In order to achieve this, consistent methodology must be used within each laboratory. However, there is a lack of information whether the methodology itself could impact the infection outcome or antibiotic efficacy.

The use of frozen stocks to infect mice is a practice that has shown, for some labs, more stable CFU counts in murine pneumonia models. However, this approach is not recommended when working with Gram-negative bacteria since they may not tolerate freeze-thaw cycles well.

### Infection procedure variables

#### Immunosuppression

Most studies used immunocompromised mice to test antimicrobial efficacy of small molecule antibiotics. The use of the neutropenic model aims to achieve a robust bacterial infectivity in mice and reflect the bacterial growth or replication observed in patients (Andes and Lepak, 2017), but it does not aim to mimic immunocompromised or neutropenic patients. Although the use of neutropenic versus naïve animals could have an effect on PK/PD target, most clinical dose predictions have been based on the neutropenic model (Andes and Lepak, 2017).

#### Induction of neutropenia

The most common protocol to achieve neutropenia in mice is intraperitoneal administration of cyclophosphamide (150 mg/kg) four days before the infection and again one day (100 mg/kg) before the infection. The use of neutropenic animals, generated with this cyclophosphamide protocol, was proposed as the standard. Some research groups use a slight variation in the cyclophosphamide protocol increasing the dose to 250 mg/kg at day minus four when working with more difficult pathogens such as *A. baumannii;* however, other groups confirmed that the proposed standard cyclophosphamide protocol allows researchers to achieve consistent and robust infectivity even when working with these bacteria.

#### Anaesthesia

Although most of the institutions employ inhalational anesthetics, it was considered that this variable should not be standardized because institutions typically already have their own approved methods for anesthetizing mice. While standardization of the type of anesthesia was not considered necessary by the panel, the depth and duration of anesthesia is an important parameter to consider. The anesthetic plane should be deep enough to enable full inhalation of the inoculum without sneezing or ‘bubbling’ on the nares, yet not so deep that respiration is slow and/or shallow. The proper depth of anesthesia will enable as much of the inoculum as possible to reach the lungs. This parameter is also important for the animal’s survival. If the anesthesia is too deep, animals may have difficulty recovering after inoculation.

#### Infection route

IT and IN are the most commonly used routes of infection. Considering that the IN route is less invasive and less technically challenging, it was proposed as the standard. The IT route requires more skill to master, but it may provide more reproducible results, particularly if it enables a greater volume of the inoculum to reach the lungs. Thus, the IT route could be considered a reasonable alternative when working with strains of low pathogenicity in mice and when sufficiently skilled personnel are available.

#### Infection volume

There is a range of inoculum volume, typically 20-50 μL, reported in the literature. The initial recommendation from COMBINE was for a standard of 50 μL for inoculation of mice. However, further discussion among the panelists raised some important points for consideration. The panel noted that this volume may be too high for inbred mice of similar age as they are often considerably smaller, which may lead to lung inflammation. In addition, some institutions have restrictions on the volume that can be administered via the IN route. Lower inoculum volumes, according to some researchers, may cause the inoculum to concentrate in a single lung lobe rather than being dispersed throughout the lungs, resulting in a highly focal infection. To address these concerns, it was decided to recommend a volume range of 20-50 L rather than a specific standard volume.

#### Infection dose

Bacterial pathogenicity studies in mice are necessary prior to choosing the bacterial strains and infectious dosage. The infectious dose required for different bacterial strains can vary widely depending on virulence and growth characteristics for a given strain. While the inoculum concentration can have a significant impact on the outcome of the study, standardization was deemed not necessary since the baseline bacterial levels in the lung will be standardized instead.

### Treatment and endpoint variables

#### Treatment starting point

In most protocols, antibiotic treatment begins 2 h post infection (p.i.) Thus, this was the standard proposed by and agreed upon by the panel experts.

#### Baseline burden

The number of bacteria present in the infected tissue at the time treatment starts (2 h p.i.) was seen as an important variable to standardize. As noted above, bacterial culture and inoculum preparation variables do not need to be standardized as long as the study achieves a consistent and harmonized level of CFU in the lungs at the start of the treatment. A standard of 6.0-6.5 log_10_ CFU at the start of the treatment was initially proposed. Several panelists expressed their concern that this range was too narrow and potentially infeasible to reliably attain, especially for large bacterial collections. Broadening the range, however, led to concerns about increased variability in study outcomes. After further discussion, a consensus was reached to use a standard range of 6-7 log_10_ CFU as the average for the group at the start of treatment.

#### Bacterial growth

This variable is important to consider, as little or no growth indicates that there is spontaneous bacterial clearance, which can make a compound appear more efficacious. Although it was suggested that 1.5-2 log_10_ CFU growth in untreated mice after 26 h.p.i may be better for efficacy studies, this can be difficult to obtain with some strains. Overall, a minimum of 1 log_10_ CFU growth was considered necessary, and this was proposed as the standard. If growth in untreated (or vehicle-treated) mice is less than 1 log_10_ CFU, then results from that study should be flagged and potentially excluded from the analysis.

#### Length of study

Most of the studies employing an acute murine pneumonia model used an endpoint of 26 h p.i., which corresponds to 24 h post initiation of treatment, and this was agreed as the consensus standard. The inclusion of more than one time point could be valuable, but it is not practical to recommend as a standard practice. When performing longer studies, i.e., over several days, the panel recommended including 26 h p.i. as one of the time points in order to be able to compare the results among studies.

#### Primary endpoint

The primary outcome most commonly used and widely accepted as an important assessment for efficacy is the bacterial burden in the lung. Therefore, CFU per lung was proposed as the standard outcome measure for small molecule antibiotic efficacy studies. Additional study outcomes such as survival, immune response, etc. may be relevant for other types of molecules or experiments; typically, these would be considered secondary endpoints.

#### Sample processing methods

There was considerable variability in the methods used for sample processing. The following was proposed as the standard: collection of whole lungs without perfusion or weighing, homogenization in saline via method and volume of choice and adjustment of readouts to report CFU per lung. The weight of the lungs does not influence the results if data are normalized.

In the reported studies, a plethora of media were used, independent of the organism isolated. It was agreed that standardizing the lung sample culturing methods was not necessary, as long as a methodology is consistently applied and samples within a study are treated the same. Comparison of growth and antimicrobial resistance patterns pre- and post-infection was not discussed during the workshop; however it could help to better characterize bacterial strains.

## Workshop survey

At the conclusion of the panel discussion, we conducted an online survey to collect the participants’ opinions on the proposed and debated standards for the murine model. The survey output is given in the Supplementary Document and summarized in Table 1. Except for the sex of the animals, most of the participants agreed with all the proposed standard variables.

## Conclusion

The use of standard protocols avoids the lengthy process of *in vivo* protocol development and reduces the variability of the results. It therefore complies with the 3R principle, reducing the number of animals required in the preclinical studies. In this report we identified variables that may have a significant impact on the results obtained and recommend harmonized standards for these variables. A single, standard protocol for conducting all murine lung infection models is not feasible, as models should always be adapted to best suit the particular question being answered. Thus, while the standard protocol proposed here is suitable for antimicrobial efficacy characterization of small molecule antibiotics against *P. aeruginosa, K. pneumoniae*, and *A. baumannii*, it may not be suitable for testing other type of molecules or other bacterial species. However, this standard protocol can serve as a “starting point” for further modification to support other types of testing. Having considered all comments and suggestions received during the workshop, the COMBINE team is developing a standard murine lung infection protocol that includes the parameters described in this report. This protocol will be used within the COMBINE project to assess preclinical efficacy of small molecule antibiotics. The aim of this future work is two-fold: 1) determine reproducibility of results from lab-to-lab using the standard protocol; and 2) improve preclinical-to-clinical translation by comparing PK/PD and efficacy results obtained using this standard protocol with clinical trial data for these benchmark antibiotics.

## Data availability statement

The original contributions presented in this study are included in the article/Supplementary material, further inquiries can be directed to the corresponding author.

## Author contributions

RA: writing—original draft. RA, BK, KH, JLH, CL, JUH, SS, SR, VA-C, DH, PG, LF, and IB-D: conceptualization, manuscript review, and editing. All authors read and approved the final manuscript.
