# Supplementary material for: Expert workshop summary: Advancing toward a standardized murine model to evaluate treatments for antimicrobial resistance lung infections
Source: Front Microbiol. 2022 Sep 8;13:988725. doi: 10.3389/fmicb.2022.988725 (PMC9493304; doi:10.3389/fmicb.2022.988725)
Supplement: Supplementary file 1 [file Data_Sheet_1.DOCX]

**Expert Workshop Summary: Advancing towards a standardized murine model to evaluate treatments for AMR lung infections**

Rakel Arrazuria, Bernhard Kerscher, Karen E. Huber, Jennifer L. Hoover, Carina Vingsbo Lundberg, Jon Ulf Hansen, Sylvie Sordello, Stephane Renard, Vincent Aranzana-Climent, Diarmaid Hughes, Philip Gribbon, Lena E. Friberg, Isabelle Bekeredjian-Ding

**Sup. Figure 1.** Detailed Workshop Agenda.

**
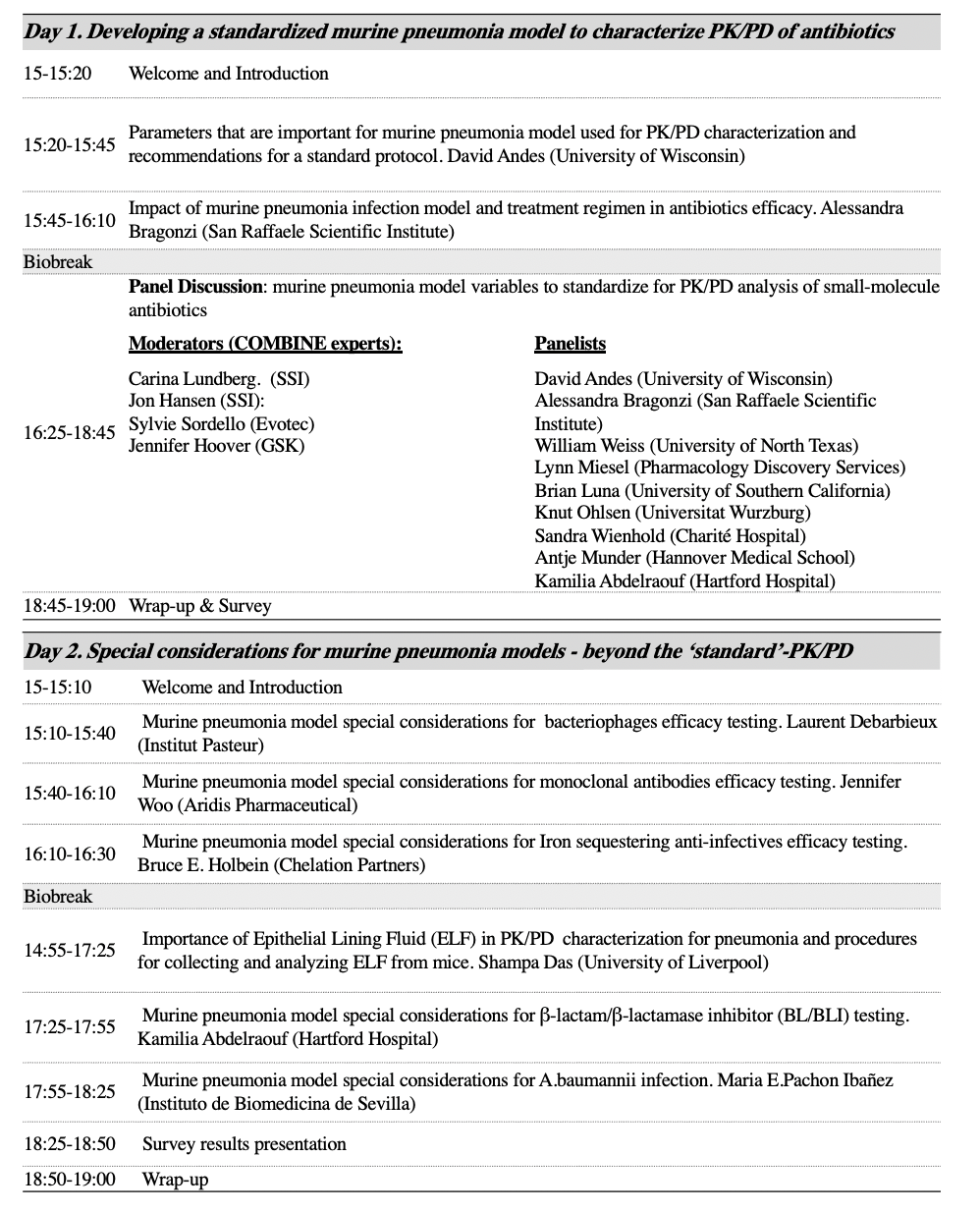
**

**SUMMARY OF THE WORKSHOP PRESENTATIONS**

**Murine pneumonia model for preclinical PK/PD assessment**

Dr. David Andes (University of Wisconsin, USA) provided an overview of the murine lung infection model for preclinical PK/PD studies and the variables affecting the results. One such variable is the starting inoculum (Andes and Lepak, 2017; Maglio et al., 2004). The use of neutropenic animals also affects bacterial growth (Drusano et al., 2018; Luna et al., 2019) and dose response results (Andes and Craig, 2002), with generally higher drug exposure required for efficacy in neutropenic vs. immunocompetent mice. Nonetheless, the use of neutropenic mice allows for testing of bacterial strains that are otherwise not sufficiently virulent to grow *in vivo*, and most clinical predictions have been based on neutropenic models. Bacterial infection can affect lung PK considerably (Crandon et al., 2009; Keel et al., 2012), and also plasma PK to a lesser extent (Bulik et al., 2017). In addition, using different bacterial species and strains can impact the PK/PD results (Andes and Lepak, 2017). The infection site may also influence the outcome (Andes and Craig, 2006; Craig et al., 2010; Lepak et al., 2012, 2017; Louie et al., 2011), hence Dr. Andes did not recommend deriving PK/PD for lung infections from the traditionally used murine thigh infection models. This highlights the importance of the murine lung infection model for translating pre-clinical results to clinical outcomes for pneumonia indications.

Dr. Alessandra Bragonzi (San Raffaele Scientific Institute, Italy) summarized the characteristics of acute and chronic murine pneumonia models for antibiotic efficacy studies against *P. aeruginosa* isolated from cystic fibrosis (CF) patients. Immunocompetent mice are usually used to study acute and chronic infections induced by bacteria isolated from CF patients. In the acute model, treatment efficacy was influenced by the class of antibiotic and the route of administration, as demonstrated by reduced efficacy of tobramycin and colistin treatment following intranasal administration compared to aerosol or subcutaneous treatments (Cigana et al., 2020). Acute and chronic murine pneumonia models aim to mimic different stages of disease, and antibiotic efficacy may differ substantially between stages (Cigana et al., 2020). Drug efficacy studies in an acute murine model may predict the efficacy for early treatment of chronic infections but may not be predictive of efficacy in late-stage treatment of chronic infections.

**Murine pneumonia models- beyond the ‘standard’ PK/PD**

Dr. Shampa Das (University of Liverpool, UK) focused on epithelial lining fluid (ELF) drug concentrations as an informative measurement in pneumonia PK/PD characterizations and the procedures for collecting and analyzing ELF from mice. She emphasized that drug concentration in the ELF is not a substitute for plasma drug concentration and that measurements in both compartments should be performed. Using ceftazidime-avibactam and meropenem-vaborbactam as an example, Dr. Das illustrated how a detailed understanding of PK/PD and penetration into the lungs may allow the utilization of existing clinical information from one indication to potentially support the initial approval for further indications. Moreover, she explained how PD targets defined in preclinical models are applied to human ELF and plasma PK data through modeling using cefepime in combination with enmetazobactam as an example. PK in both plasma and ELF were assessed and exposure targets in plasma and ELF were established (Johnson et al., 2020). Furthermore, based on a human ELF study, PK in plasma and ELF were defined through modelling using population analysis. Subsequent Monte Carlo simulations assessed the attainment of PD targets defined in preclinical models (Das et al., 2021).

Dr. Kamilia Abdelraouf (Hartford Hospital, USA) tackled specific considerations and complexities in assessing the PK/PD of beta-lactam/beta-lactamase inhibitor (BL/BLI) combinations in the murine model. As for most antibiotics, mice have a faster rate of BL/BLI elimination than humans, consequently requiring higher doses relative to the body weight compared to humans. Moreover, such two-component systems may lead to PK interactions (Asempa et al., 2019). Therefore, as for most combination treatments, it is important to assess the PK of both BL and BLI alone and in combination. It should be taken into account that altered disposition in mice does not necessarily indicate PK interaction in humans (29), especially considering the high dosage given to mice in relation to body weight. Additionally, the exposure of BLI required for β-lactamase inhibition to achieve specific efficacy endpoints may vary considerably across enzyme classes and bacterial strains (Abdelraouf et al., 2020). Dr. Abdelraouf recommended the use of a fixed BL Human Simulated Regimen (HSR) while varying exposure of the BLI component to assess PK/PD, and emphasized that studies to correlate murine ELF PK/PD indices to clinical outcome are needed.

Dr. Laurent Debarbieux (Institut Pasteur, France) focused on a murine pneumonia model for bacteriophage efficacy testing. He employed immunocompetent outbred mice and immunodeficient inbred mice, intranasally (IN) infected with bioluminescent *P. aeruginosa* or *Escherichia coli* to test the efficacy of a bacteriophage treatment (Debarbieux et al., 2010; Dufour et al., 2015). *P. aeruginosa* PAK_P1 phage therapy or *E. coli* 536_P1 phage therapy delay or even completely prevent death of infected mice in a dose dependent manner. Phage treatment was successful in wild type mice, but failed in mice lacking the Toll-like receptor signaling adaptor MyD88 ^-/-^ and in neutrophil depleted wild-type mice. This was due to the emergence of phage-resistant bacteria, suggesting an important role for the innate immune system acting in concert with the phage therapy (Roach et al., 2017). This is corroborated by mathematical models, which suggest that the number of phage-resistant bacteria increases over time when immune activation is lacking. In immunocompetent animals, the immune system does not allow the proliferation of resistant bacteria, leading to an overall successful phage treatment for both *P. aeruginosa* and *E. coli* infected mice (Delattre et al., 2021; Roach et al., 2017). The relevance of these findings, particularly those in neutrophil depleted and immunocompetent mice, has to be evaluated in humans to understand their true value. Recommendations for the murine model in bacteriophage efficacy testing include handling controls (without phages) first to prevent contamination, performing a post-analysis of bacterial clones for phage resistance, and considering the contribution of the host immune response in bacterial clearance. Alongside these studies, the use of a live imaging system has been pivotal to test and compare the efficacy of several phages with a limited number of animals (Henry et al., 2013).

Dr. Jennifer Woo (Aridis Pharmaceutical, USA) provided insights on the preclinical efficacy testing of monoclonal antibody (mAb) therapies. She highlighted the importance of using immunocompetent animals in these studies. Dr. Woo illustrated the challenges encountered in the implementation of murine pneumonia models for the assessment of mAb therapies against *A. baumannii*. Her recommended strategies to assist in the establishment of a robust infection in immunocompetent mice included adaptation of the bacterial challenge strains to the host by three successive passages; this may lead to increased pathogenicity and more robust infections in wild-type immunocompetent mice. Moreover, the addition of mucin to the inoculum and the use of the intratracheal (IT) route may further support a robust infection. Dr. Woo emphasized the relevance of consistency in animal handling, dosing, infection procedures and treatment methods in establishing a reliable animal model that provides reproducible results.

Dr. Bruce E. Holbein (Chelation Partners, Canada) shared his experience using a previously described murine pneumonia model (Harris et al., 2017) to study the efficacy of DIBI, an iron-sequestering anti-infective (Holbein et al., 2021). Immunocompetent murine models were used to allow the analysis of the inflammatory response and markers of infection. Following a single DIBI treatment, significant reductions in the bacterial burden and inflammatory cytokines in the lung and serum of DIBI treated mice were observed compared to sham-treated animals (Parquet et al., 2019). In addition, the iron withdrawal produced by DIBI treatment improved ciprofloxacin antibiotic efficacy, reducing the potential for antibacterial resistance development (Parquet et al., 2019). For testing of iron-sequestering anti-infectives, Dr. Holbein recommended assessing efficacy against strains with different pathogenicity levels, including highly pathogenic as well as less pathogenic strains, to thoroughly evaluate the effect of the anti-infective. Strains with differing degrees of virulence may display differences in their iron acquisition ability and other virulence determinants; hence evidence should be generated that the anti-infective treatment does not inadvertently support an infection with a nonvirulent strain.

Dr. María E. Pachón-Ibáñez (Instituto de Biomedicina de Sevilla, Spain) summarized the variables that may have an impact on the establishment of successful murine lung infections with Gram negative bacteria in general and *A. baumannii* in particular. Some of the relevant variables mentioned by Dr. Pachón-Ibáñez were the sex and age of the mice and the use of mucin in the inoculum. Some data suggest that female C57BL/6 mice are more susceptible to infection than males (Pires et al., 2020), and aged female C57BL/6 mice (18-21 months) have an increased susceptibility to *A. baumannii* infection (Gu et al., 2018). Mucin enhances bacterial infectivity (Harris et al., 2019), allowing a decrease in the inoculum concentration in several mouse strains (Harris et al., 2019). However, mucin also induces neutrophilic infiltration to the lung, which affects the alveolar, interstitial and bronchial areas. Therefore, it was felt that, the use of mucin in immunotherapy studies should be limited and restricted to cases where the establishment of an infection cannot be achieved by other means.

**WORKSHOP SURVEY**

**EXPERT WORKSHOP: Advancing towards a standardized murine model to evaluate treatments for AMR lung infections**

**Q1: What is your role in the workshop?**


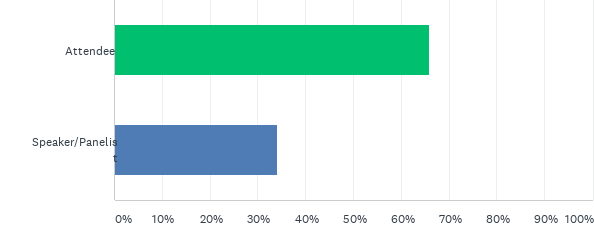


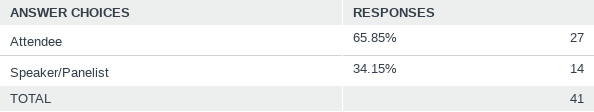


**Q2: Do you have experience with murine pneumonia models of Gram-negative pathogens?**


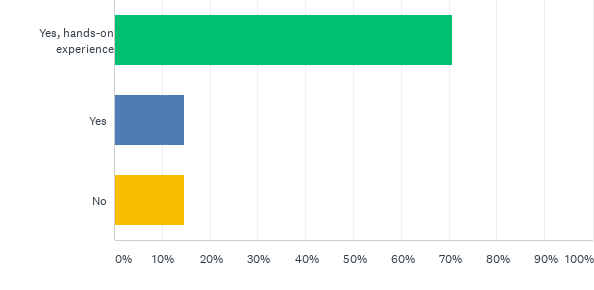


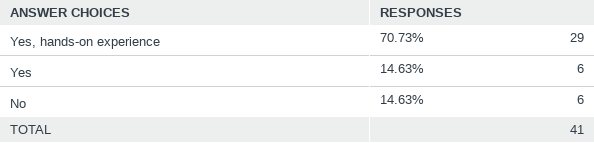


**Q3: Please, select your current affiliation:**


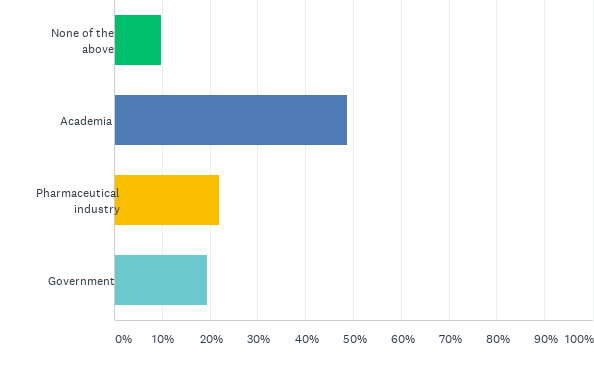


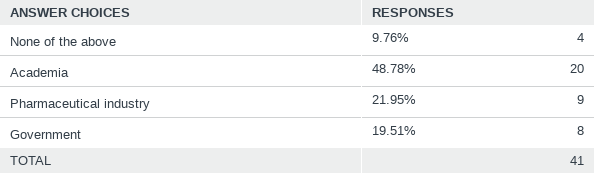


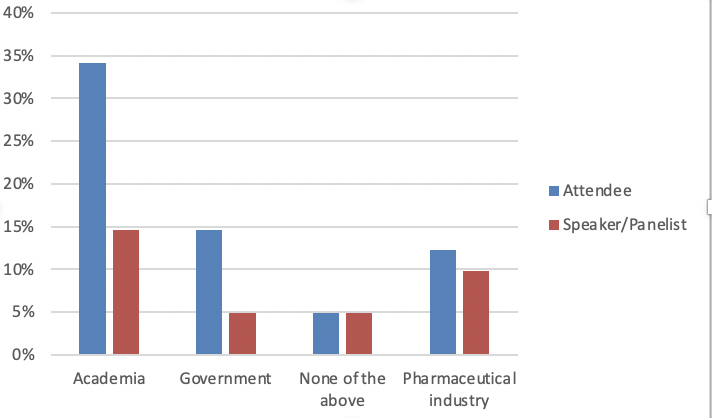


**Q4: CD1 Outbred mice should be used for neutropenic standard murine pneumonia model for PK/PD analysis of small molecule antibiotics.**


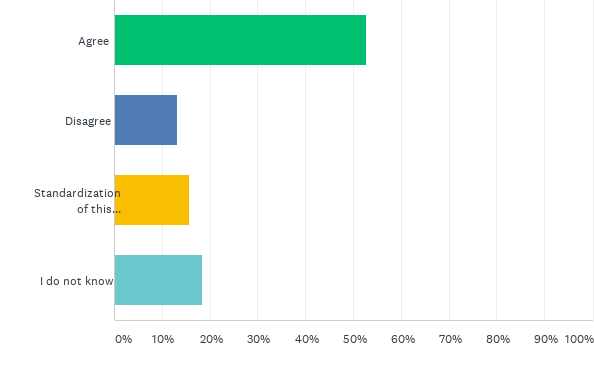


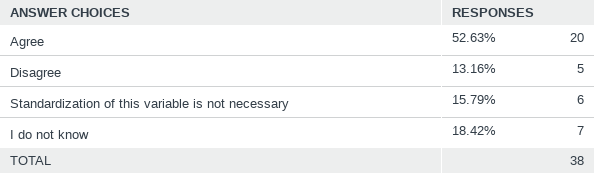


**Q5: Which mice would you recommend instead for neutropenic standard murine pneumonia model for PK/PD analysis of small molecule antibiotics?**


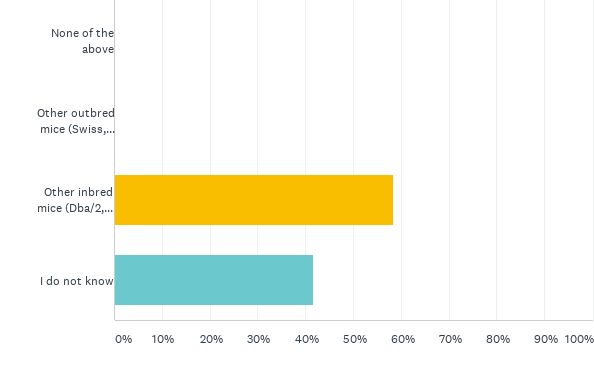


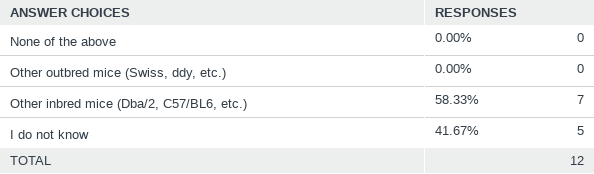


**Q6: Should only female mice be used for a standard murine pneumonia protocol?**


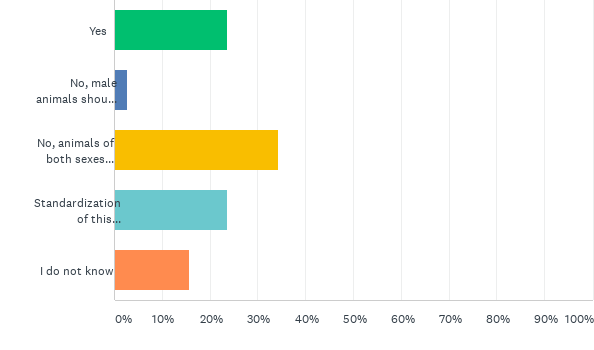


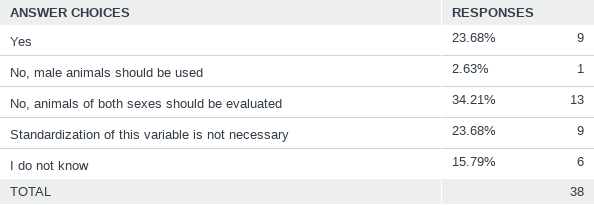


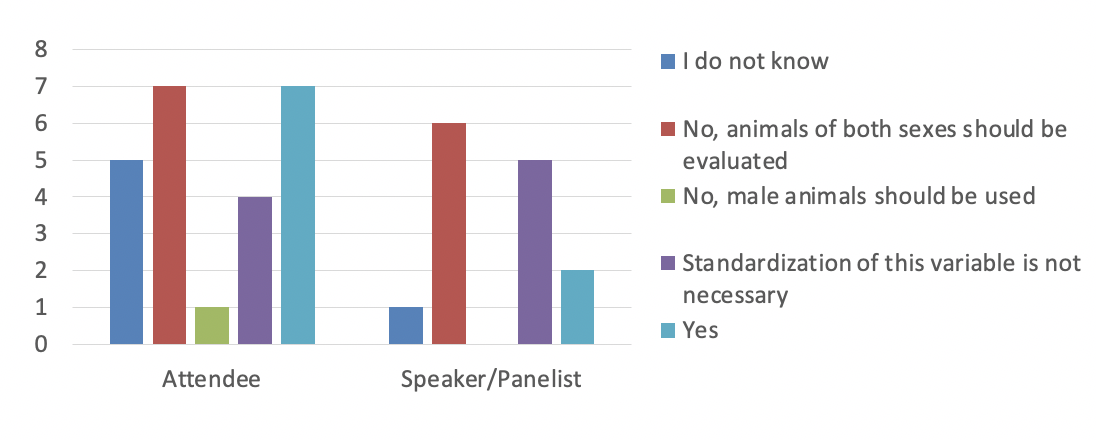


**
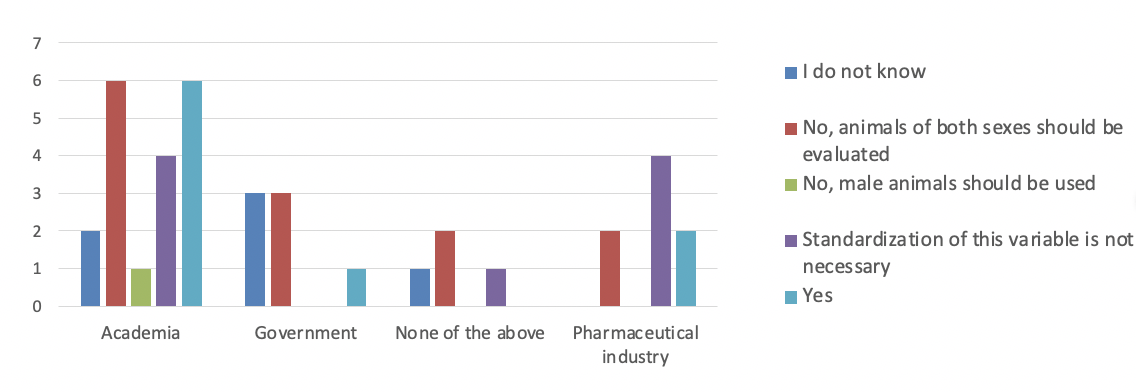
**

**Q7: Mice that are 6 ± 1 week old at the time of infection (day 0) should be used in the standard murine pneumonia protocol.**


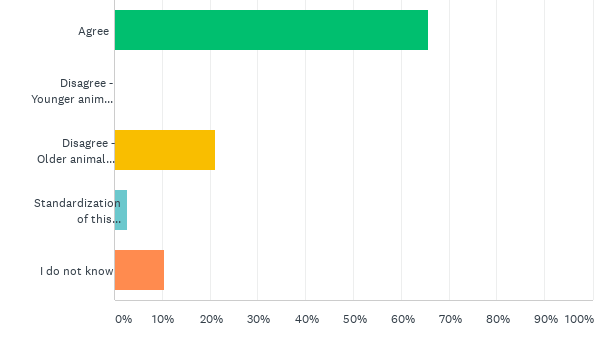


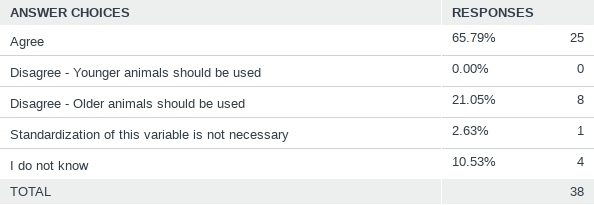


**Q8: Five to six animals per treatment group should be used for assessing efficacy in a standard murine pneumonia protocol.**


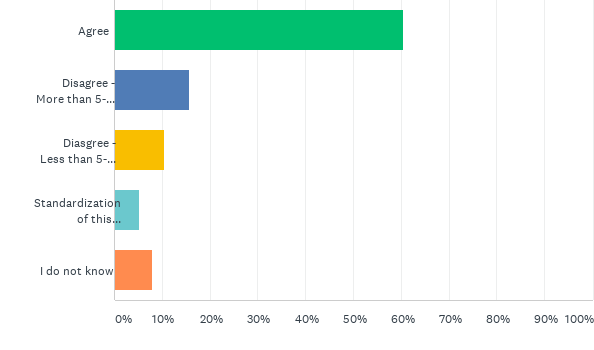


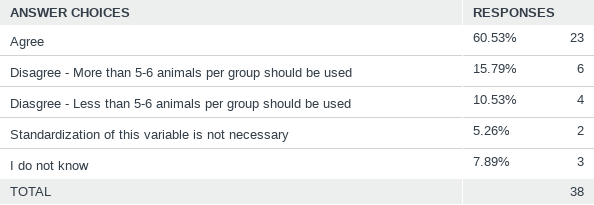


**Q9: What kind of bacterial strains should be used in a standard murine pneumonia infection model? (please select all that apply)**


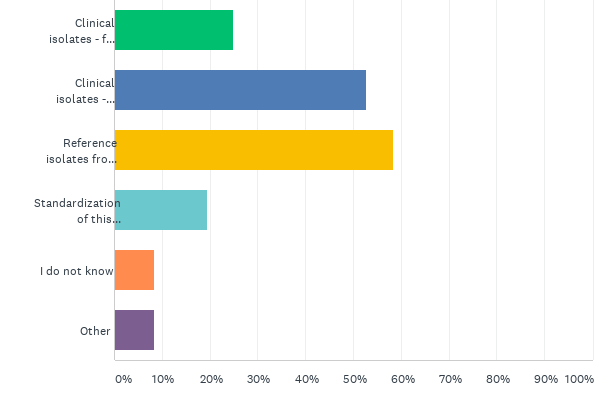


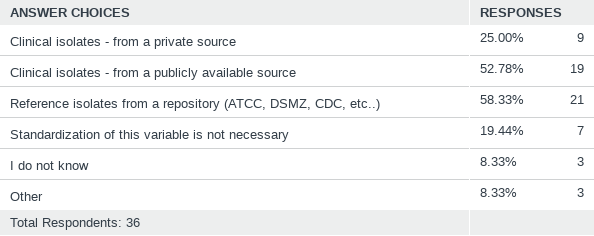


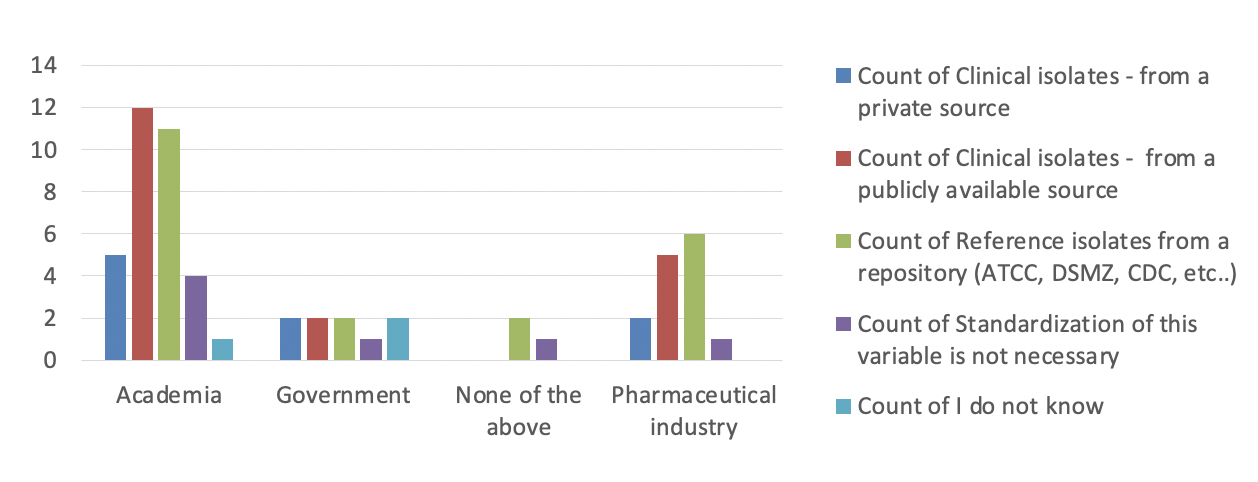


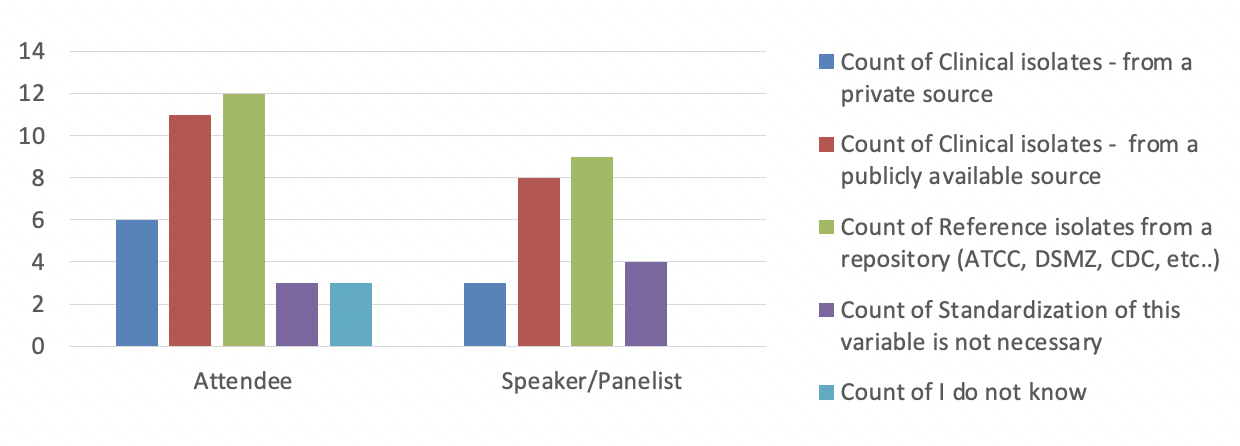


**Q10: Should strains be animal passaged prior to using them in the neutropenic murine pneumonia model?**


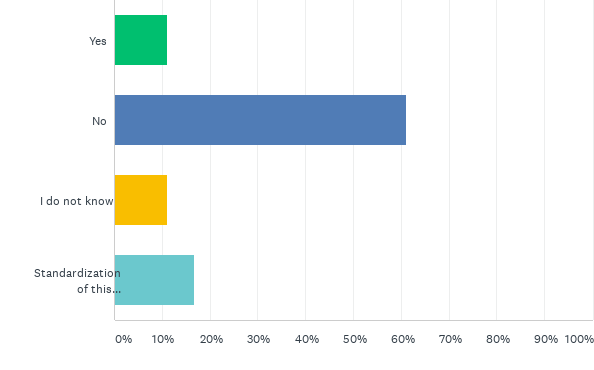


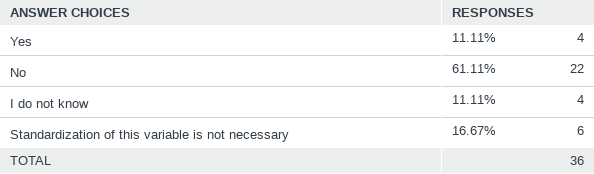


**Q11: At least one strain from a worldwide accessible strain bank should be included when assessing PK/PD in the murine pneumonia model to enable benchmarking or comparison of the results.**


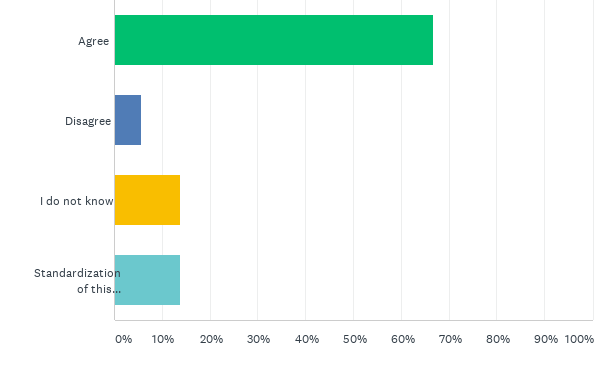


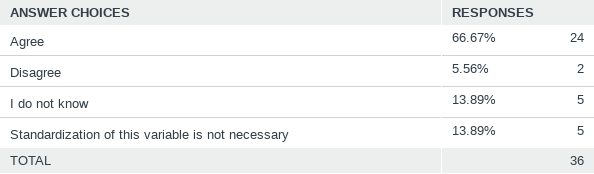


**Q12: Should a fresh bacterial culture be used for inoculating mice in the standard protocol?**


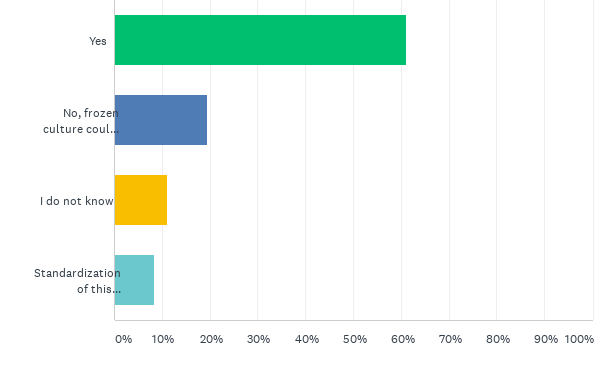


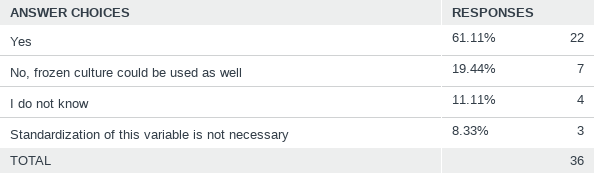


**Q13: Should the inoculum for infecting mice be prepared with bacteria in logarithmic (or exponential) phase of growth?**


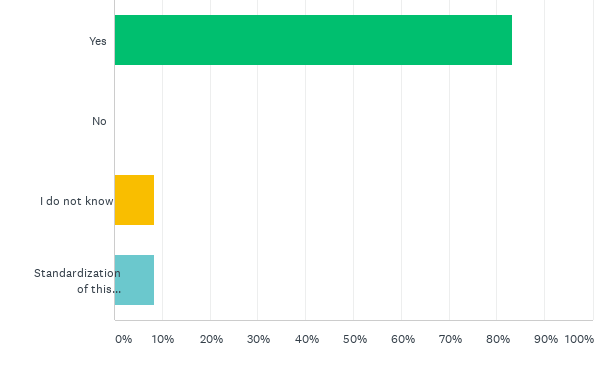


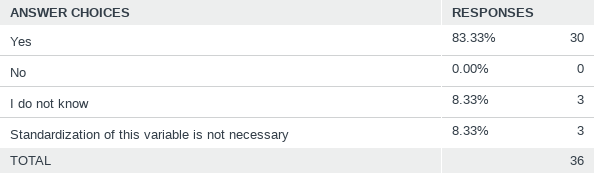


**Q14: Should cultured bacteria undergo washing steps before being used to infect mice?**


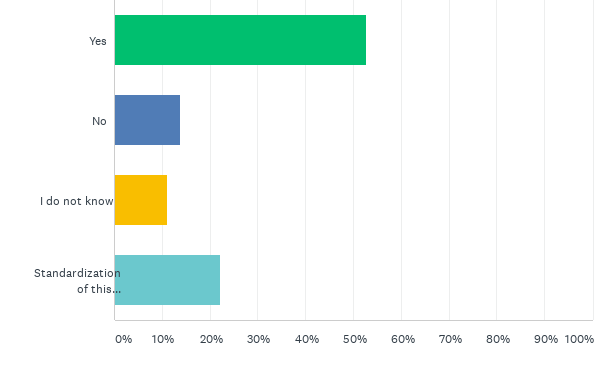


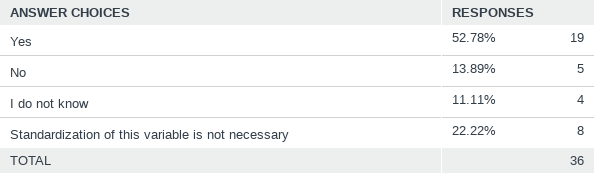


**Q15: Should cold PBS (phosphate buffered saline) be used as a vehicle for the bacterial inoculum?**


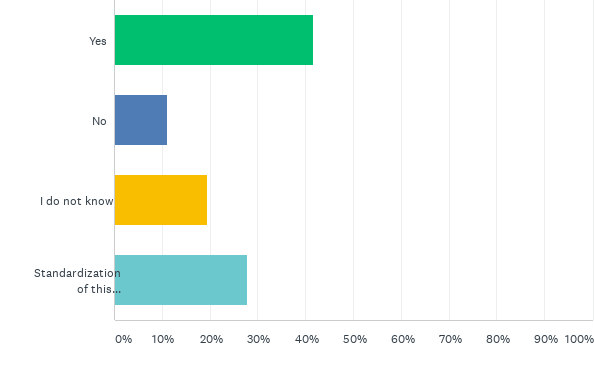


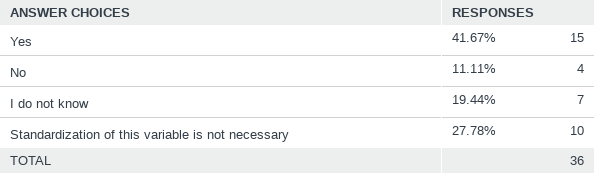


**Q16: Which vehicle would you recommend instead of cold PBS?**


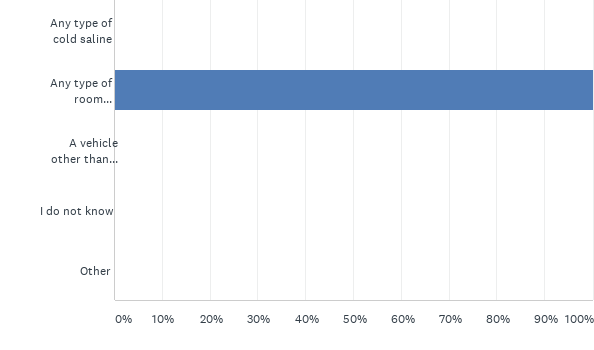


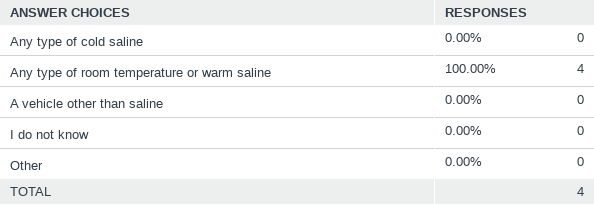


**Q17: Neutropenic mice (treated with cyclophosphamide) should be used for standard murine pneumonia model for PK/PD analysis of small molecule antibiotics.**


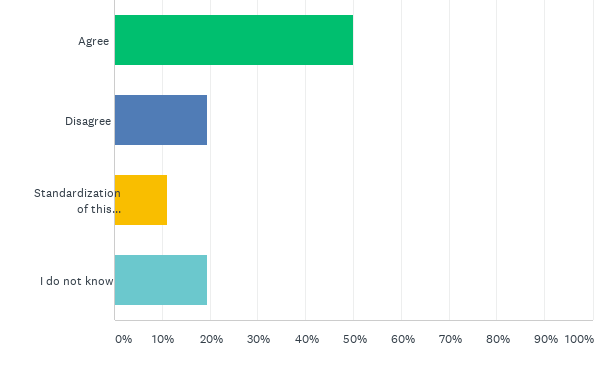


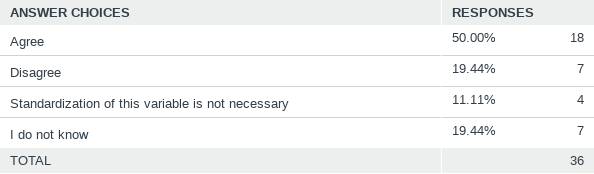


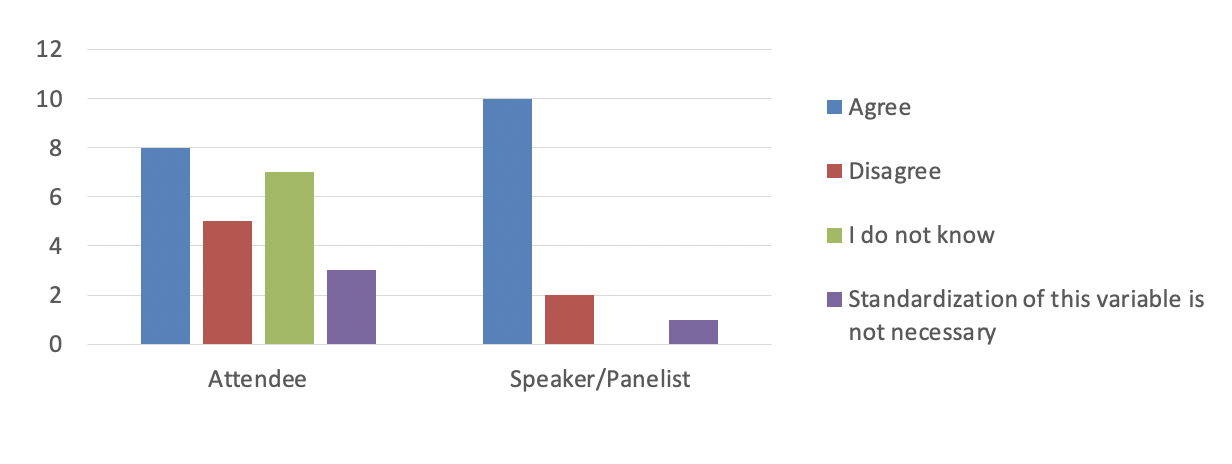


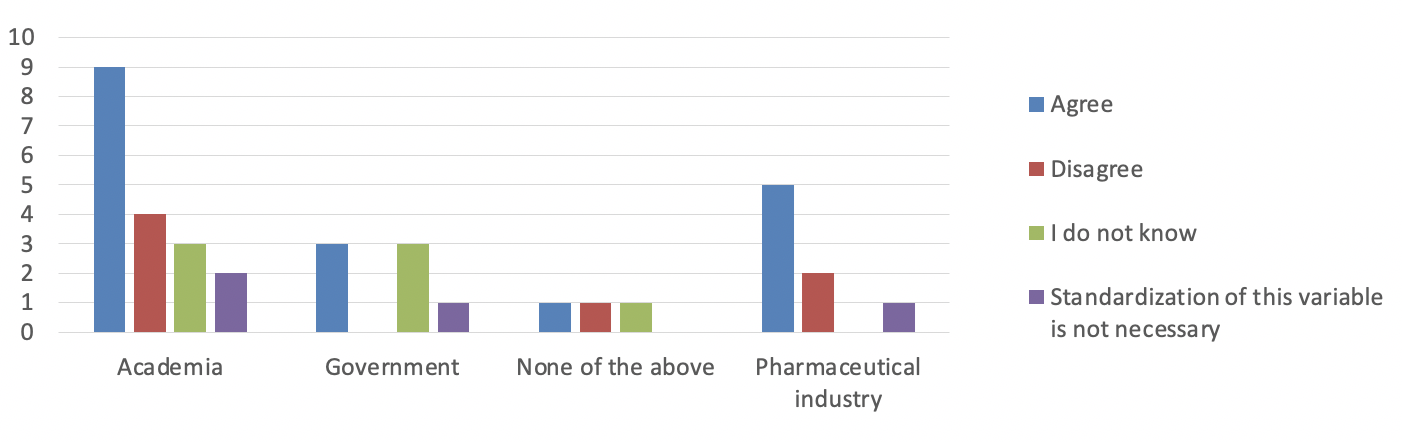


**Q18:Why do you think, neutropenic mice should not be used for standard murine pneumonia model for PK/PD analysis of small molecule antibiotics?**

- Compromised neutropenic mice may show altered characteristics for some test agents.
- My concern is about compound that can be up taken by phagocytes (e.g. macrolides).
- It does not represent the reality, in which healthy individuals will also be infected and treated with these small molecule antibiotics.
- I believe the model should mimic what happens in clinic (…).
- Immunocompetent mice can also be used.
- Mice are generally pan cytopenic, not just neutropenic. PK/PD parameters can be very different in infected v non infected mice.
- Some strains can establish successful infection in immunocompetent mice.
- Because It can impact the PK and by the way PD (since part of immune system is depleted)

**Q19: Should the standard cyclophosphamide protocol of 150 mg/kg on day -4 and 100 mg/kg on day -1 injected intraperitoneally (IP) be recommended to induce neutropenia in mice?**


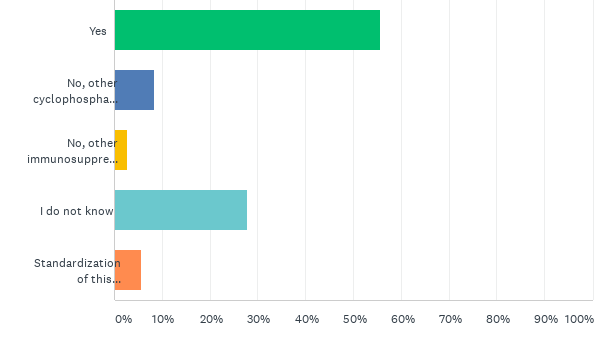


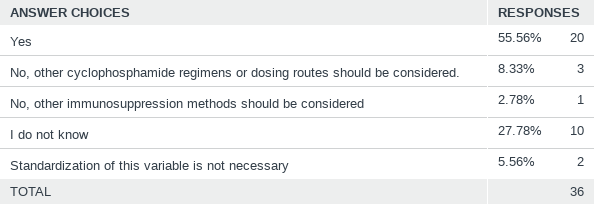


**Q20: Should the intranasal infectious route be recommended for the murine pneumonia standard protocol?**


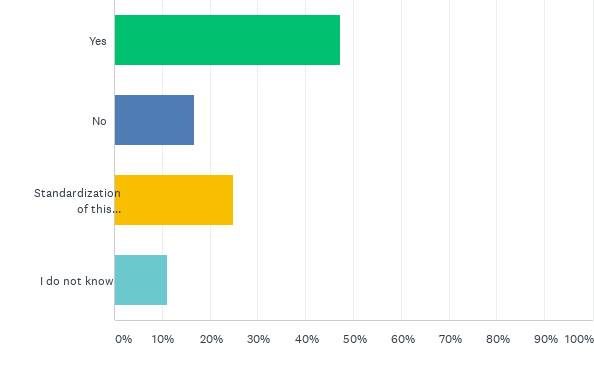


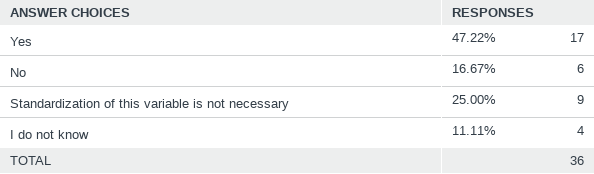


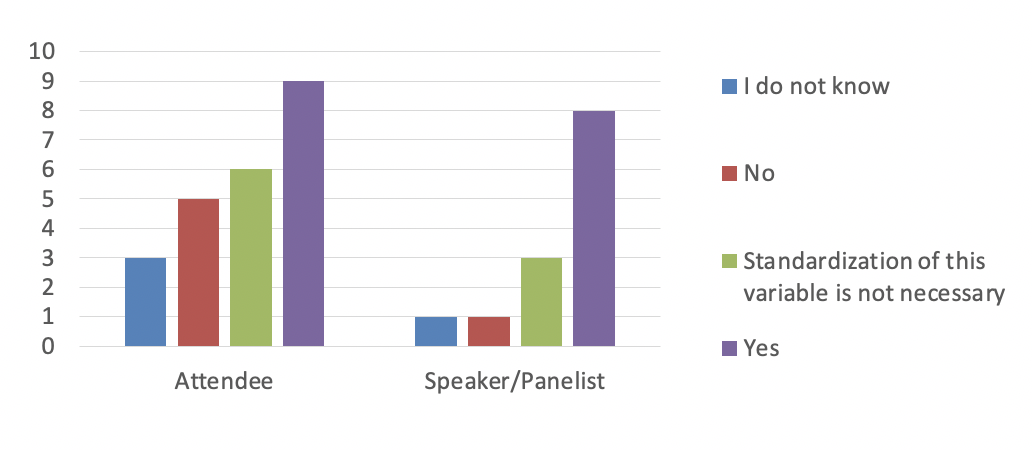


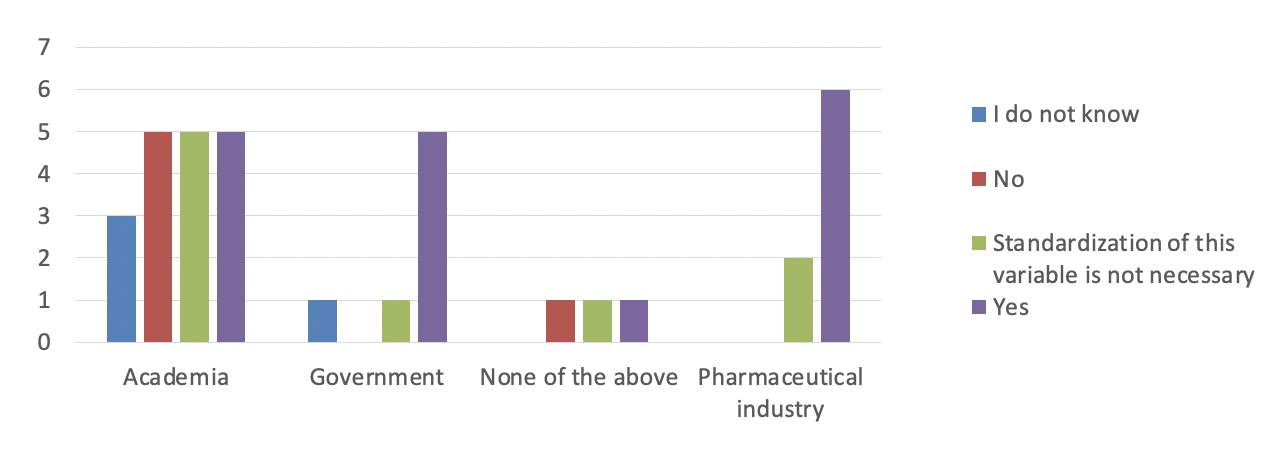


**Q21: Should a 50 µl volume of inoculum be used for the intranasal infectious route?**


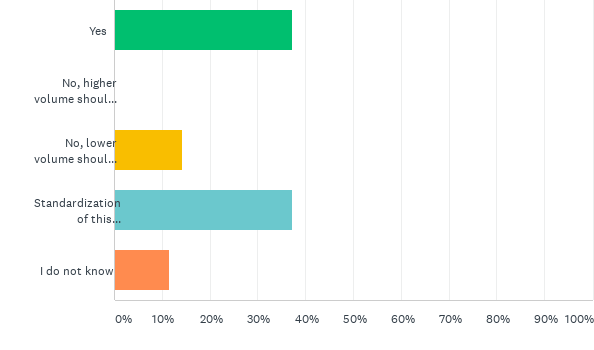


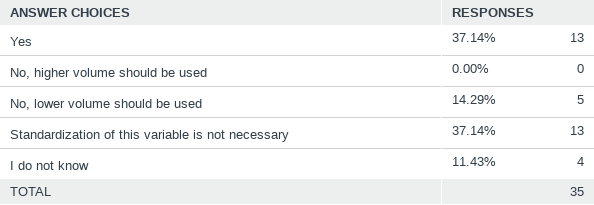


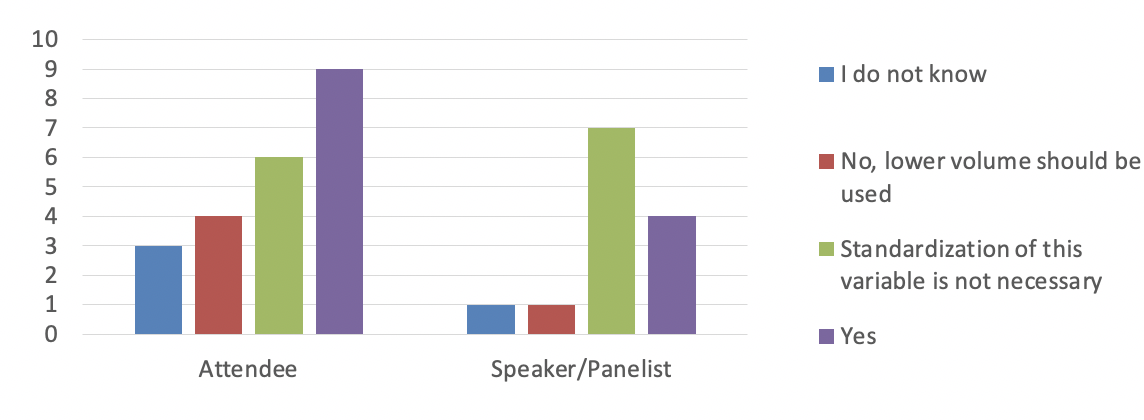


**
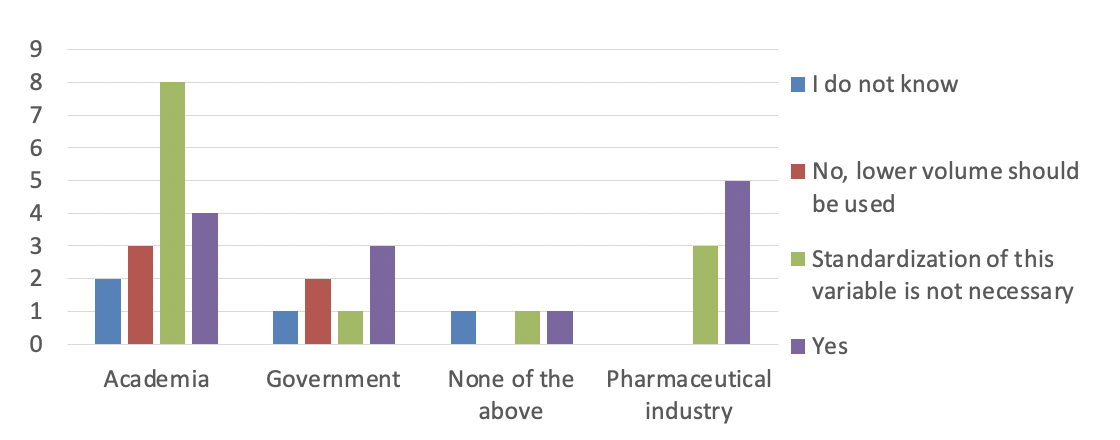
**

**Q22: Do you agree that standardization of the administered infectious dose (i.e. CFU given to the mice) is not needed considering that the baseline CFU/lung will be standardized?**


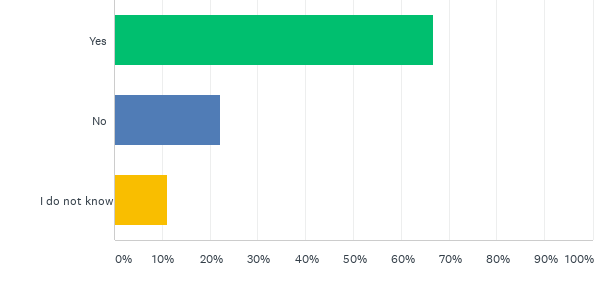


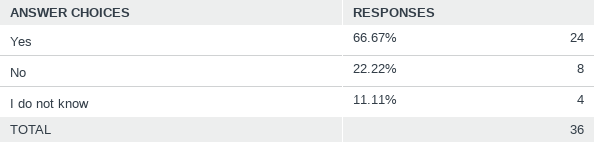


**Q23: Should antibiotic therapy start at 2h post-infection in the standard murine pneumonia model for PK/PD analysis of small molecule antibiotics.**


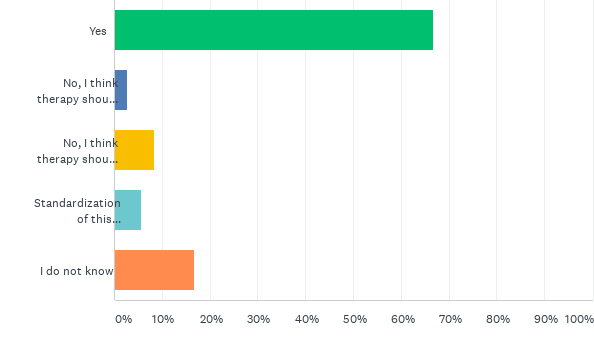


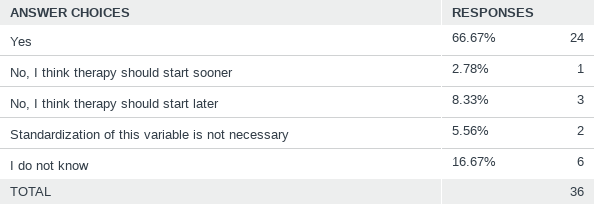


**Q24: Should the baseline bacterial burden at start of therapy aim to reach 6-6.5 (±0.2) log10 CFU/lung?**


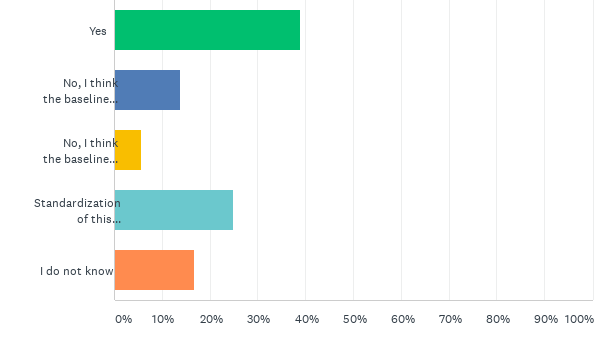


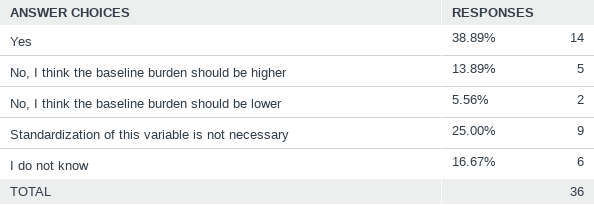


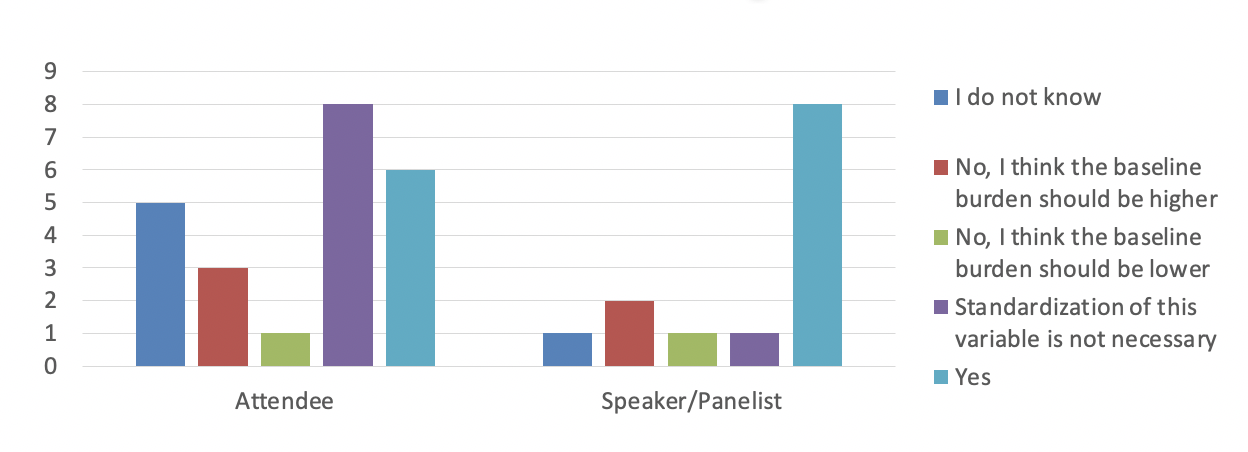


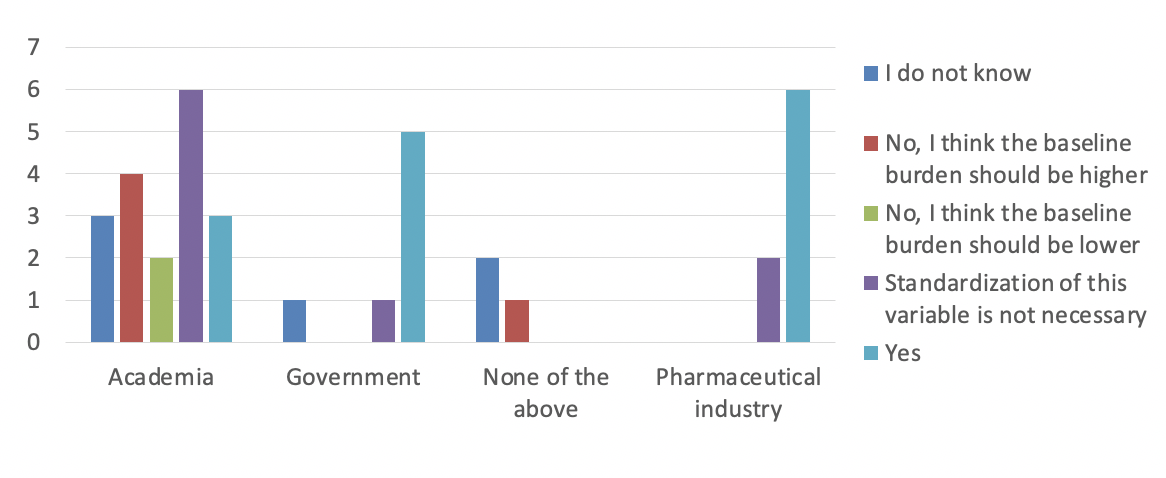


**Q25: Should the experimental endpoint in the standard murine pneumonia model for PK/PD analysis of small molecule antibiotics be at 26 h post-infection?**


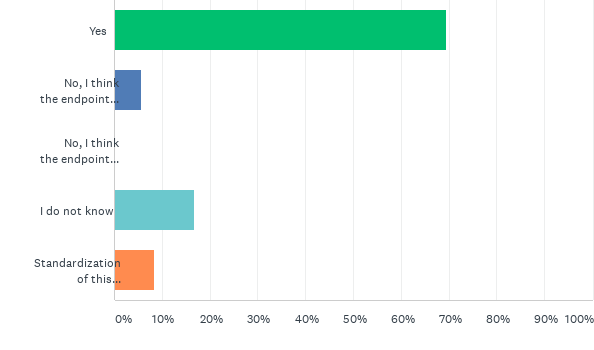


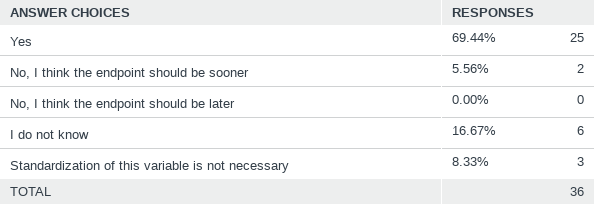


**Q26: Should a minimum of 1 log10 CFU of growth in untreated mice be recommended in the standard protocol?**


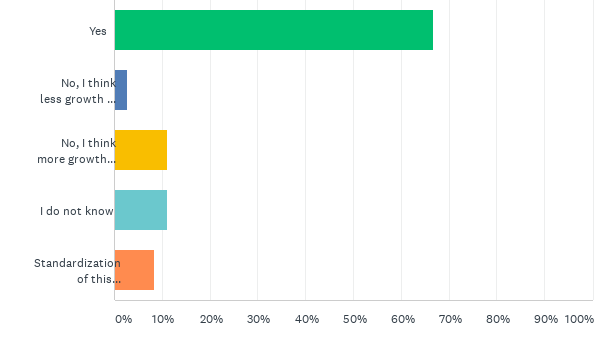


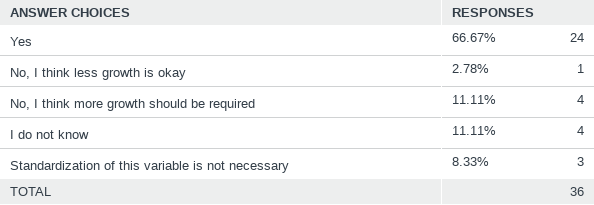


**Q27: Do you think that, in addition of bacterial CFU/lung, other parameters should also be measured to assess efficacy for PK/PD of a small molecule antibiotics?**


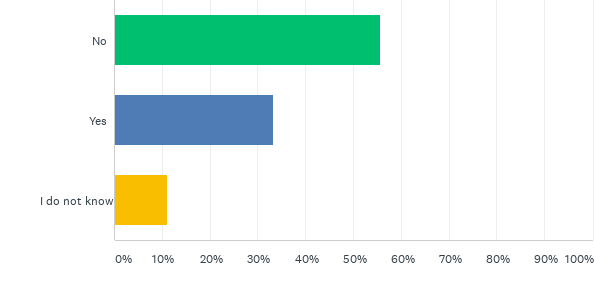


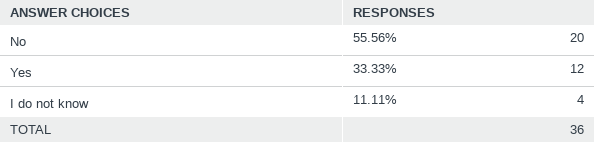


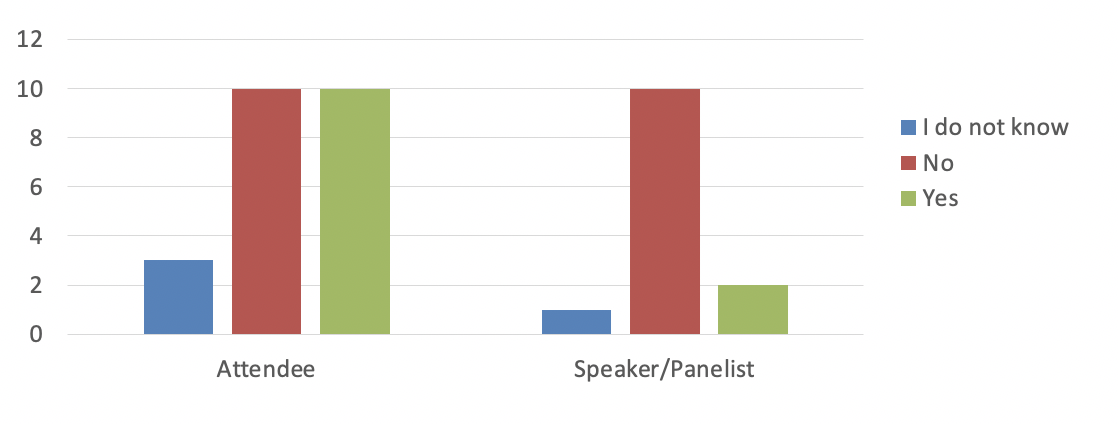


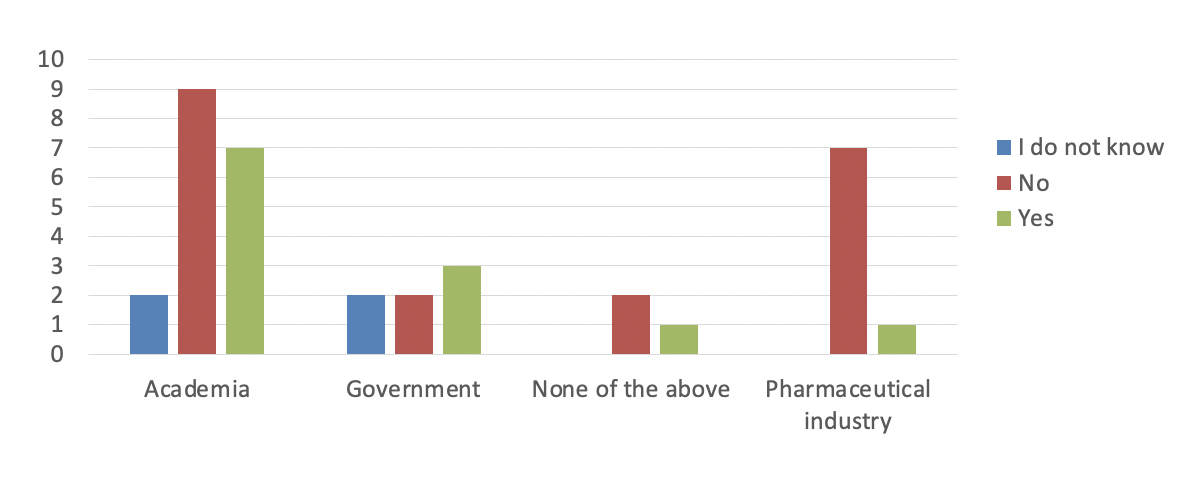


**Q28: What other parameter(s) do you think should be measured to assess efficacy for PK/PD of a small molecule antibiotics? (select all that apply)**


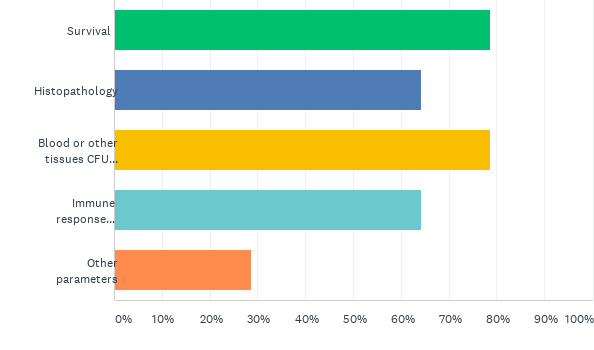


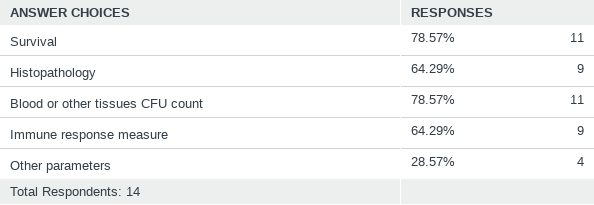


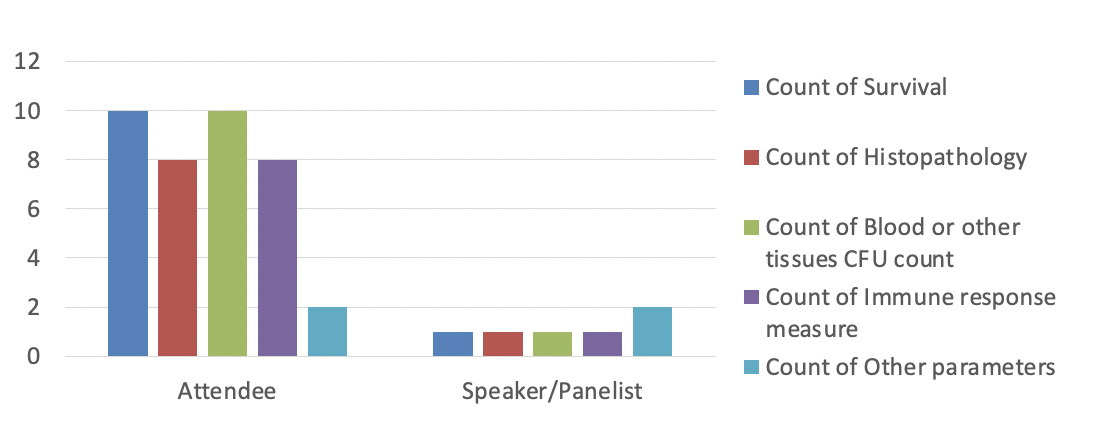


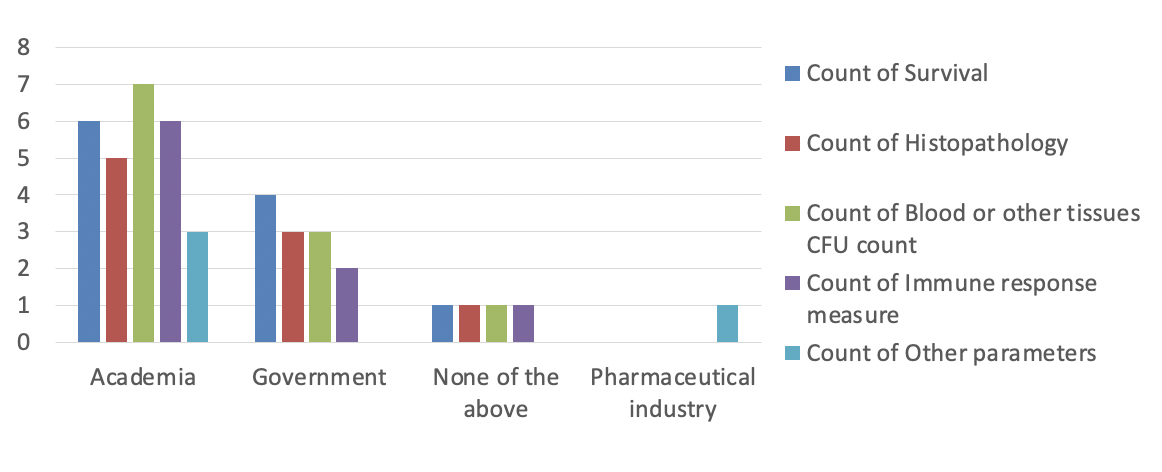


**Q29: Do you think that a standard protocol for the murine pneumonia model should aim to standardize any other variable we have not discussed during the workshop or in this survey?**


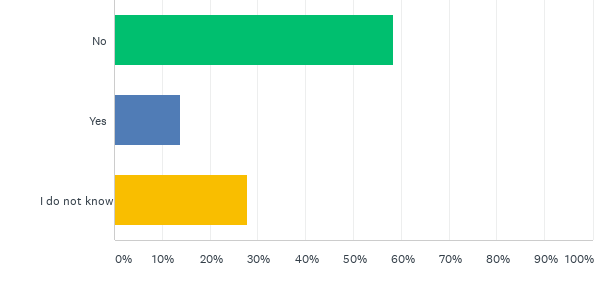


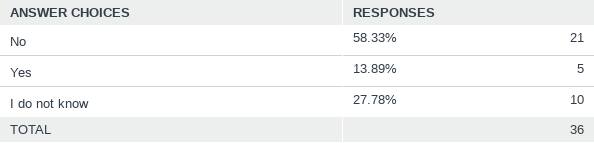


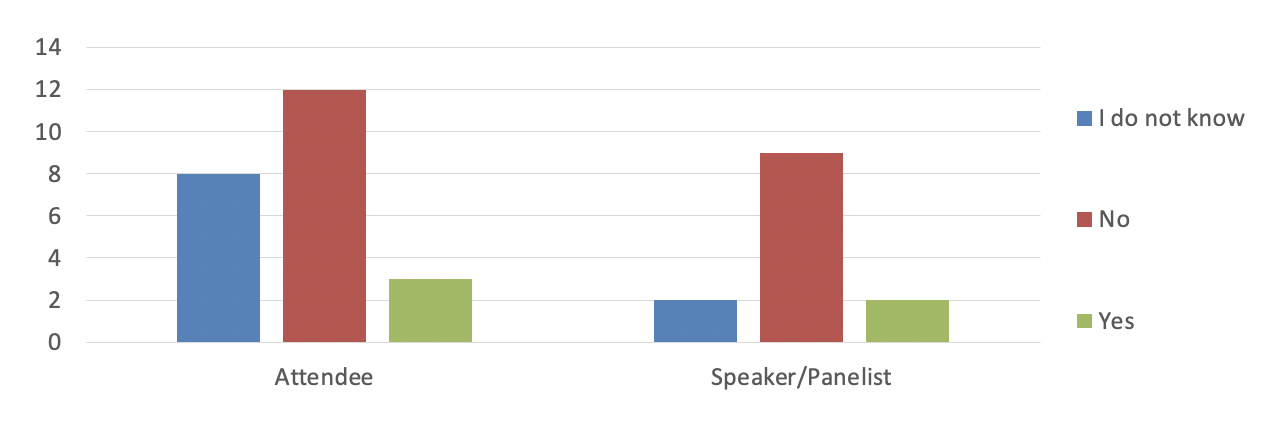


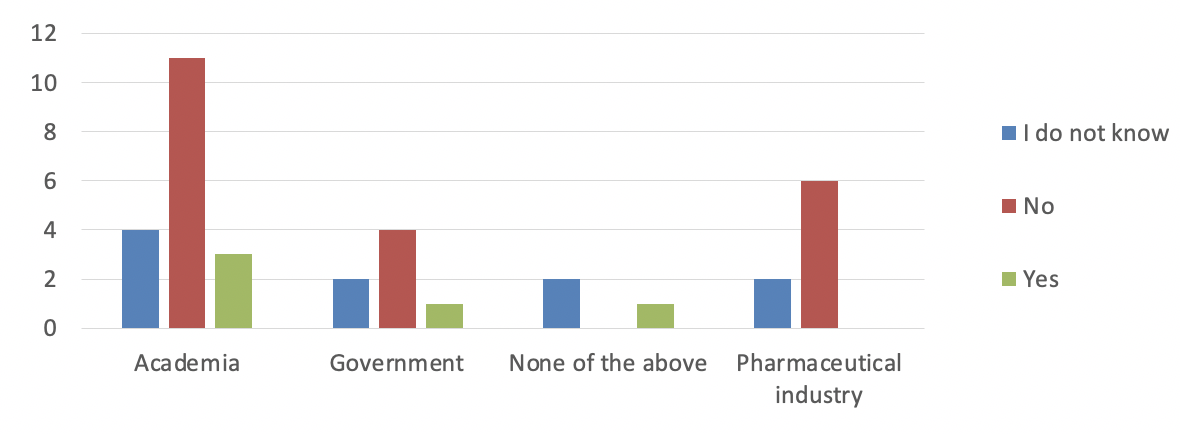


**Q30: Which other variables or parameter do you think a protocol for the murine pneumonia model should aim to standardize that we have not discussed during the workshop or in this survey?**

- Variables associated with the inoculum, treatment and endpoint: The use of porcine gastric mucin in inoculum preparation, a recommendation for a larger range of baseline CFU (6-7 +/- 0.3 log CFU/lung), standardized method of outlier data exclusion.
- Sham infected treated/ untreated control groups: circadian rhythm; when to infect, treat etc.
- Bacteria strains
- Histopathology
- Co-infections: virus infections

**REFERENCES**

Abdelraouf, K., Almarzoky Abuhussain, S., and Nicolau, D. P. (2020). In vivo pharmacodynamics of new-generation β-lactamase inhibitor taniborbactam (formerly VNRX-5133) in combination with cefepime against serine-β-lactamase-producing Gram-negative bacteria. *J. Antimicrob. Chemother.* 75, 3601–3610. doi:10.1093/jac/dkaa373.

Andes, D., and Craig, W. A. (2002). Animal model pharmacokinetics and pharmacodynamics: A critical review. Elsevier doi:10.1016/S0924-8579(02)00022-5.

Andes, D., and Craig, W. A. (2006). Pharmacodynamics of a new cephalosporin, PPI-0903 (TAK-599), active against methicillin-resistant *Staphylococcus aureus* in murine thigh and lung infection models: Identification of an in vivo pharmacokinetic-pharmacodynamic target. *Antimicrob. Agents Chemother.* 50, 1376–1383. doi:10.1128/AAC.50.4.1376-1383.2006.

Andes, D. R., and Lepak, A. J. (2017). In vivo infection models in the pre-clinical pharmacokinetic/pharmacodynamic evaluation of antimicrobial agents. Elsevier Ltd doi:10.1016/j.coph.2017.09.004.

Asempa, T. E., Motos, A., Abdelraouf, K., Bissantz, C., Zampaloni, C., and Nicolau, D. P. (2019). Efficacy of human-simulated epithelial lining fluid exposure of meropenem-nacubactam combination against class A serine -lactamase-producing Enterobacteriaceae in the neutropenic murine lung infection model. *Antimicrob. Agents Chemother.* 63. doi:10.1128/AAC.02382-18.

Bulik, C. C., Okusanya, Ó. O., Lakota, E. A., Forrest, A., Bhavnani, S. M., Hoover, J. L., et al. (2017). Pharmacokinetic-pharmacodynamic evaluation of gepotidacin against gram-positive organisms using data from murine infection models. *Antimicrob. Agents Chemother.* 61. doi:10.1128/AAC.00115-16.

Cigana, C., Ranucci, S., Rossi, A., De Fino, I., Melessike, M., and Bragonzi, A. (2020). Antibiotic efficacy varies based on the infection model and treatment regimen for *Pseudomonas aeruginosa*. *Eur. Respir. J.* 55. doi:10.1183/13993003.02456-2018.

Craig, W. A., Andes, D. R., and Stamstad, T. (2010). In vivo pharmacodynamics of new lipopeptide MX-2401. *Antimicrob. Agents Chemother.* 54, 5092–5098. doi:10.1128/AAC.00238-10.

Crandon, J. L., Kim, A., and Nicolau, D. P. (2009). Comparison of tigecycline penetration into the epithelial lining fluid of infected and uninfected murine lungs. *J. Antimicrob. Chemother.* 64, 837–839. doi:10.1093/jac/dkp301.

Das, S., Fitzgerald, R., Ullah, A., Bula, M., Collins, A. M., Mitsi, E., et al. (2021). Intrapulmonary pharmacokinetics of cefepime and enmetazobactam in healthy volunteers: Towards new treatments for nosocomial pneumonia. *Antimicrob. Agents Chemother.* 65. doi:10.1128/AAC.01468-20.

Debarbieux, L., Leduc, D., Maura, D., Morello, E., Criscuolo, A., Grossi, O., et al. (2010). Bacteriophages can treat and prevent *Pseudomonas aeruginosa* lung infections. *J. Infect. Dis.* 201, 1096–1104. doi:10.1086/651135.

Delattre, R., Seurat, J., Haddad, F., Nguyen, T.-T., Gaborieau, B., Kane, R., et al. (2021). Combination of in vivo phage therapy data with in silico model highlights key parameters for treatment efficacy. *bioRxiv*, 2021.03.04.433924. doi:10.1101/2021.03.04.433924.

Drusano, G. L., Corrado, M. L., Girardi, G., Ellis-Grosse, E. J., Wunderink, R. G., Donnelly, H., et al. (2018). Dilution factor of quantitative bacterial cultures obtained by bronchoalveolar lavage in patients with ventilator-associated bacterial pneumonia. *Antimicrob. Agents Chemother.* 62. doi:10.1128/AAC.01323-17.

Dufour, N., Debarbieux, L., Fromentin, M., and Ricard, J.-D. (2015). Treatment of Highly Virulent Extraintestinal Pathogenic *Escherichia coli* Pneumonia With Bacteriophages*. *Crit. Care Med.* 43, e190–e198. doi:10.1097/CCM.0000000000000968.

Gu, H., Liu, D., Zeng, X., Peng, L. S., Yuan, Y., Chen, Z. F., et al. (2018). Aging exacerbates mortality of *Acinetobacter baumannii* pneumonia and reduces the efficacies of antibiotics and vaccine. *Aging (Albany. NY).* 10, 1597–1608. doi:10.18632/aging.101495.

Harris, G., Holbein, B. E., Zhou, H., Howard Xu, H., and Chen, W. (2019). Potential mechanisms of mucin-enhanced *Acinetobacter baumannii* virulence in the mouse model of intraperitoneal infection. *Infect. Immun.* 87. doi:10.1128/IAI.00591-19.

Harris, G., KuoLee, R., Xu, H. H., and Chen, W. (2017). Mouse Models of *Acinetobacter baumannii* Infection. *Curr. Protoc. Microbiol.* 46, 6G.3.1-6G.3.23. doi:10.1002/cpmc.36.

Henry, M., Lavigne, R., and Debarbieux, L. (2013). Predicting in vivo efficacy of therapeutic bacteriophages used to treat pulmonary infections. *Antimicrob. Agents Chemother.* 57, 5961–5968. doi:10.1128/AAC.01596-13.

Holbein, B. E., Ang, M. T. C., Allan, D. S., Chen, W., and Lehmann, C. (2021). Iron-withdrawing anti-infectives for new host-directed therapies based on iron dependence, the Achilles’ heel of antibiotic-resistant microbes. *Environ. Chem. Lett.* 19, 2789–2808. doi:10.1007/s10311-021-01242-7.

Johnson, A., McEntee, L., Farrington, N., Kolamunnage-Dona, R., Franzoni, S., Vezzelli, A., et al. (2020). Pharmacodynamics of cefepime combined with the novel extended-spectrum-lactamase (ESBL) inhibitor enmetazobactam for murine pneumonia caused by ESBLproducing *Klebsiella pneumonia*. *Antimicrob. Agents Chemother.* 64. doi:10.1128/AAC.00180-20.

Keel, R. A., Crandon, J. L., and Nicolau, D. P. (2012). Pharmacokinetics and pulmonary disposition of tedizolid and linezolid in a murine pneumonia model under variable conditions. *Antimicrob. Agents Chemother.* 56, 3420–3422. doi:10.1128/AAC.06121-11.

Lepak, A. J., Marchillo, K., Pichereau, S., Craig, W. A., and Andes, D. R. (2012). Comparative pharmacodynamics of the new oxazolidinone tedizolid phosphate and linezolid in a neutropenic murine Staphylococcus aureus pneumonia model. *Antimicrob. Agents Chemother.* 56, 5916–5922. doi:10.1128/AAC.01303-12.

Lepak, A. J., Zhao, M., and Andes, D. R. (2017). Comparative pharmacodynamics of telavancin and vancomycin in the neutropenic murine thigh and lung infection models against Staphylococcus aureus. *Antimicrob. Agents Chemother.* 61. doi:10.1128/AAC.00281-17.

Louie, A., Liu, W., Kulawy, R., and Drusano, G. L. (2011). In vivo pharmacodynamics of torezolid phosphate (TR-701), a new oxazolidinone antibiotic, against methicillin-susceptible and methicillin-resistant *Staphylococcus aureus* strains in a mouse thigh infection model. *Antimicrob. Agents Chemother.* 55, 3453–3460. doi:10.1128/AAC.01565-10.

Luna, B. M., Yan, J., Reyna, Z., Moon, E., Nielsen, T. B., Reza, H., et al. (2019). Natural history of *Acinetobacter baumannii* infection in mice. *PLoS One* 14, e0219824. doi:10.1371/journal.pone.0219824.

Maglio, D., Ong, C., Banevicius, M. A., Geng, Q., Nightingale, C. H., and Nicolau, D. P. (2004). Determination of the in vivo pharmacodynamic profile of cefepime against extended-spectrum-beta-lactamase-producing Escherichia coli at various inocula. *Antimicrob. Agents Chemother.* 48, 1941–1947. doi:10.1128/AAC.48.6.1941-1947.2004.

Parquet, M. del C., Savage, K. A., Allan, D. S., C Ang, M. T., Chen, W., Logan, S. M., et al. (2019). Antibiotic-Resistant *Acinetobacter baumannii* Is Susceptible to the Novel Iron-Sequestering Anti-infective DIBI in Vitro and in Experimental Pneumonia in Mice. *Antimicrob. Agents Chemother.* 63. doi:10.1128/AAC.00855-19.

Pires, S., Peignier, A., Seto, J., Smyth, D. S., and Parker, D. (2020). Biological sex influences susceptibility to *Acinetobacter baumannii pneumonia* in mice. *JCI Insight* 5. doi:10.1172/JCI.INSIGHT.132223.

Roach, D. R., Leung, C. Y., Henry, M., Morello, E., Singh, D., Di Santo, J. P., et al. (2017). Synergy between the Host Immune System and Bacteriophage Is Essential for Successful Phage Therapy against an Acute Respiratory Pathogen. *Cell Host Microbe* 22, 38-47.e4. doi:10.1016/j.chom.2017.06.018.
